# Drought Tolerance Strategies and Autophagy in Resilient Wheat Genotypes

**DOI:** 10.3390/cells11111765

**Published:** 2022-05-27

**Authors:** Kahleen Hickey, Magnus Wood, Tom Sexton, Yunus Sahin, Taras Nazarov, Jessica Fisher, Karen A. Sanguinet, Asaph Cousins, Helmut Kirchhoff, Andrei Smertenko

**Affiliations:** 1 Institute of Biological Chemistry, Washington State University, 1772 NE Stadium Way, P.O. Box 99163, Pullman, WA 99164, USA; kathleen.hickey@wsu.edu (K.H.); magnus.wood@wsu.edu (M.W.); yunus.sahin@wsu.edu (Y.S.); taras.nazarov@wsu.edu (T.N.); jessica.fisher@wsu.edu (J.F.); 2 School of Biological Sciences, Washington State University, P.O. Box 644236, Pullman, WA 99164, USA; thomas.sexton@wsu.edu (T.S.); acousins@wsu.edu (A.C.); 3 Department of Crop and Soil Sciences, Washington State University, P.O. Box 646420, Pullman, WA 99164, USA; karen.sanguinet@wsu.edu

**Keywords:** drought, peroxisomes, autophagy, photoprotection, tolerance, wheat

## Abstract

Drought resiliency strategies combine developmental, physiological, cellular, and molecular mechanisms. Here, we compare drought responses in two resilient spring wheat (*Triticum aestivum)* genotypes: a well-studied drought-resilient Drysdale and a resilient genotype from the US Pacific North-West Hollis. While both genotypes utilize higher water use efficiency through the reduction of stomatal conductance, other mechanisms differ. First, Hollis deploys the drought escape mechanism to a greater extent than Drysdale by accelerating the flowering time and reducing root growth. Second, Drysdale uses physiological mechanisms such as non-photochemical quenching (NPQ) to dissipate the excess of harvested light energy and sustain higher F_v_/F_m_ and ϕ_PSII_, whereas Hollis maintains constant NPQ but lower F_v_/F_m_ and ϕ_PSII_ values. Furthermore, more electron donors of the electron transport chain are in the oxidized state in Hollis than in Drysdale. Third, many ROS homeostasis parameters, including peroxisome abundance, transcription of peroxisome biogenesis genes *PEX11* and *CAT*, catalase protein level, and enzymatic activity, are higher in Hollis than in Drysdale. Fourth, transcription of autophagy flux marker *ATG8.4* is upregulated to a greater degree in Hollis than in Drysdale under drought, whereas relative ATG8 protein abundance under drought stress is lower in Hollis than in Drysdale. These data demonstrate the activation of autophagy in both genotypes and a greater autophagic flux in Hollis. In conclusion, wheat varieties utilize different drought tolerance mechanisms. Combining these mechanisms within one genotype offers a promising strategy to advance crop resiliency.

## 1. Introduction

Wheat, *Triticum aestivum*, plays an important role in the human diet by providing both protein and calories [1]. Although domestication resulted in the breeding of wheat varieties with high productivity in arid climate zones [2], recent extreme weather patterns have exposed the vulnerability of dryland farming to drought. For example, the productivity of wheat farms in Washington state dropped by 30–60% due to 30% lower precipitation in 2014 and 2015. Current climate change trends predict drought to be one of the key limiting factors for wheat production globally [3,4]. In addition, drought stress often occurs in combination with other stresses, including heat and disease, which can exacerbate the yield loss [5,6]. Breeding drought-tolerant varieties is essential for food security.

Drought tolerance, in terms of maintaining yield, remains an elusive trait, even with the advancement of molecular and genomic breeding. This is due to quantitative genetic control with many small-contributing loci, inconsistent quantitative trait loci (QTL), strong genotype x environment interactions, and low heritability [7,8]. Genomic-wide association studies (GWAS) can identify and map candidate genes to be used as breeding markers with greater precision [9,10,11,12]. Therefore, expanding the list of genetic markers for physiological, morphological, and molecular survival mechanisms is essential for breeding drought-resilient varieties. 

There are three universal mechanisms of drought survival in plants [13]. The first is escaping drought by accelerating the flowering [14]. The second mechanism is drought avoidance via water-use efficiency or increasing soil moisture access. Avoidance uses a range of mechanisms, such as a bigger root system to capture moisture at deeper soil levels or stomata closure to reduce transpiration [13]. The third mechanism is drought tolerance. This mechanism focuses on withstanding dehydration through the production of protective molecules [15], which, amongst other roles, contribute to preventing the accumulation of reactive oxygen species (ROS) [16,17]. 

While ROS are produced under normal environmental conditions [16,18,19,20], the production of ROS increases under stress [21,22,23]. The main ROS are singlet oxygen (^1^O_2_), superoxide radical (O_2_^−^), hydroxyl radical (HO^.^), and hydrogen peroxide (H_2_O_2_). The accumulation of ROS causes oxidative damage to key biological molecules, including nucleic acids, lipids, and proteins [18,24,25], collectively known as oxidative stress. Oxidative stress compromises stress recovery and can trigger cell death [24,26,27,28]. As such, oxidative damage contributes to the overall loss of plant productivity under all stresses, including drought [29,30].

One of the main sources of ROS under drought is the excess of captured light energy that is not utilized for carbon dioxide fixation. Singlet oxygen can be generated by photosystem II (PSII) due to inefficient energy transfer between chlorophyll and PSII, leading to photoinhibition [27,31,32,33]. Superoxide anion radical and hydrogen peroxide can both be generated by the chloroplast electron transport chain (ETC). Superoxide radicals can also be generated at multiple sites, including photosystem I (PSI) and PSII. Hydrogen peroxide is predominantly produced during photosynthesis and photorespiration [34,35], of which very high rates are produced in the peroxisome [34,36].

Plants prevent oxidative stress using enzymatic ROS scavengers and non-enzymatic antioxidants [23,30,37]. Each cellular compartment has a specific set of ROS scavengers [23,34,38]. Amongst the most common enzymatic scavengers are superoxide dismutase (SOD), catalase (CAT), and peroxidases] [24,30,39,40]. The antioxidant group includes carotenoids, tocopherols, flavonoids, polyamides, proline, monosaccharides, ascorbate, and glutathione, amongst others [18,22,24,35,37,41,42]. 

Many studies demonstrate that the activation of the ROS scavenging system increases in response to environmental stresses. Superoxide dismutase, catalase, ascorbate peroxidase, and glutathione reductase become more active under drought-stressed conditions in wheat [22,43,44,45,46]. Enzymes of the Ascorbate-Glutathione cycle become upregulated in response to drought and salinity in wheat [42,47,48]. Drought activates the transcription and translation of ROS scavenging genes, including catalase and superoxide dismutase [43,49]. The activation of ROS scavenging pathways is shown to correlate with drought tolerance in wheat [50,51,52,53], sorghum [54,55,56], rice [57,58,59,60], tomato [61,62], and maize [63,64,65,66]. Thus, maintaining steady ROS levels (robust ROS homeostasis) is an essential drought tolerance trait. 

The activity of ROS homeostasis could be assessed indirectly by measuring the abundance of peroxisomes [67]. It was shown that ROS production in peroxisomes under drought is balanced by higher activity of peroxisomal ROS-scavenging enzymes, including CAT, ascorbate peroxidase, and SOD [18,39,40,51,68]. Peroxisome abundance increases in response to environmental stresses including light [69,70,71], ozone [72], salt [73,74], jasmonic acid [75,76], heat [77], drought [78], and heavy metals [79]. Furthermore, greater peroxisome abundance correlates negatively with yield under drought in wheat [78] and quinoa [77]. 

Peroxisomes can form de novo from the endoplasmic reticulum or proliferate through fission from existing peroxisomes [80,81,82]. Fission occurs in three stages: elongation, constriction, and fission [69,81,83]. PEROXIN11 (PEX11) proteins promote fission through peroxisome elongation–tubulation [84,85]. In addition, DYNAMIN-RELATED PROTEIN3 (DRP3) and FISSION1 (FIS1) execute the fission of peroxisomes [86,87,88]. Drought [78], heat [77], salt stress [73,89], hypoxia, and biotic stresses [90], wounding and H_2_O_2_ [91], upregulate the transcription of peroxisome fission genes.

Another process that controls peroxisome abundance is pexophagy, a specific type of autophagy that is responsible for degrading damaged or oxidized peroxisomes under both normal and stress conditions [92,93,94]. ATG8 proteins are used as a general marker of autophagy, and ATG8 is also implicated in pexophagy [92,95]. Autophagy could also contribute to drought tolerance [96,97,98].

This work aims at determining how autophagy and ROS homeostasis function in the context of photoprotection and other drought tolerance mechanisms in two drought-tolerant wheat genotypes, Drysdale and Hollis. Drysdale is hard white spring wheat bred for water use efficiency under drought conditions [99]. Hollis is hard red spring wheat [100] selected for maintaining high yield (29 bushels per acre) in locations with annual precipitation below 12” (http://smallgrains.wsu.edu/variety/; last accessed 14 May 2022). While Drysdale performs better under drought by increasing water use efficiency, the mechanism of drought tolerance in Hollis remains unknown. We found that different mechanisms contribute to drought tolerance in Drysdale and Hollis.

## 2. Materials and Methods

### 2.1. Drought Stress Treatment

Plants were grown until Zadoks stage 25 in a greenhouse or a growth chamber as specified for each experiment below [100,101]. Drought stress was induced by withholding watering. The volumetric water content (VWC) was measured using ProCheck Soil Moisture Probe with a 5TC probe (Decagon now METER Environment, Pullman, WA, USA). Once the VWC values reached below 0.2%, plant material was collected for biochemical assays. Statistical analysis was conducted using Student *t*-tests and ANOVA. 

### 2.2. Measuring the Impact of Drought on Plant Development

The greenhouse growth conditions were 60% humidity, 16/8 h light/dark cycle, 22 °C during the day, and 18 °C at night. Seeds were germinated on peat plugs for 2 weeks. The seedlings were planted together with the peat plugs into 55 gallons of U-line bins filled with Sungro 6 peat moss potting soil and watered daily. This type of soil provides the best contrast for root imaging. Fertilizer was not used. The position of bins in the greenhouse was randomized. Each bin contained one root imaging tube and two soil moisture probe tubes. Each bin was populated with five seedlings of the same genotype. Five bins were set up per each genotype: 2 well-watered controls and 3 drought treatments. The bins were watered for 1 week following transplanting the seedlings, and then the watering of the drought-stress bins was stopped. Soil moisture values were recorded twice per week in both tubes at the bottom of the bin (80 cm) and 40 cm above the bottom using a PR2 Soil Moisture Profile Probe and HH2 Soil Moisture Meter (Delta-T Devices, Cambridge, UK). Three readings per depth and each tube were collected and averaged. Root images were recorded with a CI-600 In situ Root Imager (CID Bio-Sciences, Camas, WA, USA). The images were analyzed using RootSnap! image analysis software (CID Bio-Sciences, Camas, WA, USA). To assess the size of the root system, we measured two parameters: total root length and total root count. Yield parameters including tiller number, grain number, and total yield were collected at maturity.

### 2.3. Measuring the Impact of Drought on Photosynthetic Parameters Using Phenomics Platform

The dynamic impact of drought on photosynthesis was measured in the Phenomics Facility at Washington State University. Seeds were germinated on peat plugs for 2 weeks and then transplanted into 54 × 38 cm trays filled with Sungro6 Sunshine Mix #1. Five trays were used for drought treatment, and two trays were used for watered control. The position of the trays was randomized. Each tray destined for drought stress was populated with 15 seedlings, and each of the watered control trays was populated with 35 seedlings. Seedlings were acclimated in the phenomics chamber for 5 days with daily watering, a 16/8 h light/dark cycle, 22 °C during the day and 18 °C at night, 60% humidity, and artificial illumination ~470 µmol/m^2^/s. The VWC in soil was measured using Decagon Devices Em-50 soil moisture data-logger probes (METER Environment, Pullman, WA, USA). One soil moisture probe connected to a data logger was used per each tray. Soil volumetric water content values were logged every 6 h. Drought was induced by withholding watering after seedlings were acclimated in the phenomics chamber for five days. Every day of the drought stress treatment, leaf material was collected from one plant per each drought stress tray making 5 biological replicates per genotype and five randomly selected plants from the watered control. The bottom third part of each leaf was flash-frozen in liquid nitrogen and stored at −80 °C for biochemical assays. The rest of the plant was cut at the below-ground level using scissors and discarded.

Chlorophyll fluorescence images were collected once per night using a combination of 455 nm and 630 nm saturation light and 630 nm measurement pulse using the Fluorcam XYZ system equipped with a Fluorcam 2701 LU camera (PSI Co., Drasov, Czech Republic). For these measurements’ plants were illuminated for 300 s with actinic light of 200 μmol quanta m^−2^ s^−1^ prior to taking the measurements. The images were processed by the Fluorcam 7 software (PSI Co., Drasov, Czech Republic) to derive the following parameters: F_v_/F_m_ (fluorescence quantum yield), ϕ_PSII_ (quantum yield of photosystem II photochemistry) determined after the 300 s light period), non-photochemical quenching (NPQ, determined after the 300 s light period), and NPQ components: energy-dependent quenching qE, photoinhibition qI, and a fraction of open PSII centers qL [102,103].

### 2.4. Measuring the Impact of Drought on Photosystem I

Analysis of photosystem I donor and acceptor sides was performed using a custom-built flash-spectrophotometer [104]. Seeds were planted in 1 gallon pots with Sungro6 Sunshine Mix #1 and grown in a chamber with a 16 h/8 h light/dark cycle, 22 °C during the day and 18 °C at night, 60% humidity, and light intensity ~1000 µmol/m^2^/s. Each pot contained 3–4 seedlings. Once plants reached a tillering stage (Zadok stage 25), drought was induced by withholding watering. Photosystem I measurements were performed on 4 biological replicates for each genotype and treatment when the VWC in drought-stressed pots reached 0%.

Plants were adapted to actinic light (300 μmol quanta⋅m^−2^⋅s^−1^) for five minutes and then for 60 s to each of the following light intensities 50, 100, 200, 300, 500, 800, and 1600 μmol/m^−2^/s^−1^. The electron flow through photosystem I was measured by saturating multiple turnover light pulse (100 ms)-induced redox changes of P700 (determined as the difference between the 810 nm and 900 nm absorbance change). The efficiency of ϕI was derived from P_m_′–P [105], where P is P700^+^ level for a given light intensity; P_m_′, P700^+^ maximal level in the multiple turnover pulse; P_m_, maximal P700^+^ level for the dark-adapted state (determined by a multiple turnover pulse of dark-adapted leaves); and P_0_, fully reduced P700 determined in a 0.5-s dark interval followed directly after the multiple turnover pulse. The non-photochemical loss due to oxidized donors and non-photochemical loss due to reduced acceptors were derived using P–P_0_ for donor side limitations and P_m_–P_m_′ for acceptor limitations. 

### 2.5. Measuring Peroxisome Abundance

Peroxisome abundance was measured with a small fluorescent probe Nitro-BODIPY (N-BODIPY) following a previously published procedure [73]. A 2 cm fragment of the leaf basal part was transferred into deep 96-well plates immersed in a liquid nitrogen bath and ground to a fine powder using a tissue grinder (TissueLyser II, Qiagen, Venlo, The Netherlands). Total leaf protein was extracted using 0.8 mL of the extraction buffer A (EBA; 20 mM Tris HCl, pH7.4, 500 mM NaCl, 7M Urea) by rotating the plates for 1 h. The debris was cleared by centrifugation at 3000× *g* for 30 min. Then, 20 μL of the extract was added to 80 μL of freshly prepared 2 μM solution of N-BODIPY and 100 μL of water in 96-well plates and incubated for 10 min. The fluorescence intensity was measured at 490 nm excitation wavelength and 530 nm emission wavelength using a Synergy Neo B spectrofluorometer (Biotek Instrument, Inc., Winooski, VA, USA). Five biological replicates (individual plants) with three technical replicates were performed per genotype and treatment. The background was measured as (i) 20 μL of each protein extract in 180 μL of water; and (ii) 20 μL of N-BODIPY supplemented with 180 μL of water per each 96-well plate. Both background values were subtracted from the N-BODIPY fluorescence signal value. The fluorescence intensity was normalized by the protein concentration measured with the Bradford Reagent (Biorad Laboratories, Hercules, CA, USA; Tokyo, Japan) using a calibration curve constructed with solutions of known concentration of Bovine Serum Albumin. Fluorescence intensity was calculated in arbitrary units per 1 mg of protein.

### 2.6. Microscopy

The basal 1 cm fragment of flag leaf was incubated for 15 min in a freshly prepared 1 μM solution of N-BODIPY in distilled water. The images were acquired using a Leica SP8 confocal laser scanning microscope (Leica, Wetzlar, Germany) set in the resonant scanning mode 12,000 Hz, 512 × 512 image resolution, and eight averages. Peroxisome density was calculated on 1 μm-thick optical sections taken through the cortical cytoplasm using the Fiji image analysis package (http://fiji.sc/Fiji; last accessed on 1 June 2019).

### 2.7. Measuring Activity of ROS Scavenging Enzymes

The sampling was performed at the tillering stage (Zadoks scale 18–19). A 2 cm long fragment at the flag leaf base from three individual plants was sampled and mixed in one tube. One set of three plants constituted a biological replicate. The leaf material was ground in liquid nitrogen using a mortar and pestle. Total protein extract was prepared from 150 mg of the leaf powder in 0.05 M potassium phosphate buffer (pH 7.8) supplemented with the following protease inhibitors: 200 μM AEBSF, 100 μM PMSF, 10 μM leupeptin, and 10 μM pepstatin. Protein concentration in the extract was measured using Bradford Reagent (Biorad Laboratories, Hercules, CA, USA) and a calibration curve constructed with known concentrations of Bovine Serum Albumin [106]. The enzymatic activity of CAT was measured by the rate of hydrogen peroxide decomposition at OD_240_ [107].

Ascorbate peroxidase (APX) activity was quantified in total protein extract prepared with buffer containing 0.05 M potassium phosphate buffer (pH 7.0), 5 mM EDTA, and 17 mM ascorbic acid [108]. The enzymatic activity was measured by the rate of oxidized ascorbate production at OD_290_. Guaiacol peroxidase (GPX) activity was quantified by homogenizing total protein extract with 0.05 M potassium phosphate buffer (pH 7.0) containing 1% guaiacol solution [109]. The enzymatic activity was quantified by the rate of tetratguaiacol production at OD_470_. SOD activity was quantified by homogenizing total protein extract in the buffer containing 0.154% (*w*/*v*) nitro-blue tetrazolium chloride, 5.82% (*w*/*v*) methionine, and 0.0015% (*w*/*v*) riboflavin [110]. The reaction was initiated by illuminating the cuvettes with 15 W fluorescent light for ~12 min. Absorbance was measured at OD_560_. One unit of the enzyme activity is equivalent to 50% inhibition of formazan formation and is expressed in arbitrary units.

### 2.8. Analysis of Gene Expression

Homoeologs for *CAT1* and *CAT2* and *PEX11-C* were identified using BLAST with the wheat genome database IWGSC RefSeq v2.1. Homoeologs were classified according to relative homology scores. The 3 homoeologous genes were aligned, and qRT-PCR primers were designed for regions with a sufficient number of non-conserved base pairs for capturing specific homoeologs (Supplemental Table S1). 

Total RNA was extracted from leaf using an RNeasy plant kit (Qiagen, Hilden, Germany). The leaf material was sampled as described above. cDNA was synthesized using the Maxima H Minus First Strand cDNA Synthesis Kit (Thermo Fisher Scientific, Waltham, MA, USA). Each qPCR primer was designed to target all three homoeologs as reported previously [78]. The primers are listed in Supplemental Table S1. qRT-PCR reactions were performed using Fast SYBR™ GreenMaster Mix (Thermo Fisher Scientific) in 96-wells plates on a ViiA 7 Real-Time PCR System with default ViiA™ 7 SYBR conditions. Reactions were replicated 3 times and analyzed in QuantStudio™ Real-Time PCR Software v1.3., transcription levels were normalized to housekeeping gene RNase L inhibitor-like protein [111]. 

### 2.9. RNA-Seq Analysis

To determine the impact of drought stress on the transcription of peroxisome and autophagy genes, published RNA-seq datasets for drought-stress experiments from the Gene Expression Omnibus (GEO, http://www.ncbi.nlm.nih.gov/geo/, last accessed 20 February 2020) were downloaded. Experiments were identified using the keywords “drought stress” AND “species name” [organism]. GEO accession numbers of the studies are represented in Supplemental Table S2. Five considerations were followed for selecting the RNA-Seq data: (1) more than one replicate per treatment; (2) data for the control and drought stress for each experiment; (3) RNA was extracted from the above-ground organs; (4) drought was induced by withdrawing the watering; and (5) the plants were wild type. RNA-seq datasets that satisfy these criteria were identified for four diverse species: *Arabidopsis thaliana*, *Zea mays*, *Oryza sativa*, and *Sorghum bicolor.* The analysis of responses in diverse species allows the identification of the most conserved gene transcription responses to drought.

The read quality of FASTQ files was attained using FastQC software ver. 0.11.9 (https://www.bioinformatics.babraham.ac.uk/projects/fastqc/; last accessed on 20 February 2020). The adapter sequence, low-quality bases, and short reads were trimmed using Trimmomatic ver. 0.39 [112]. Fastq files were screened to the level of Q30 and length > 50 bases. Hisat2 ver. 2.1.0 was used to map the reads to the reference genomes [113]. *A.* *thaliana* reads were mapped to the TAIR10 genome (www.arabidopsis.org; last accessed 14 April 2022); *Z.* *mays* reads were mapped to the B73v4 genome (https://www.maizegdb.org; last accessed 20 February 2020); *O.* *sativa* reads were mapped to the MSU7 genome (http://rice.plantbiology.msu.edu; last accessed 20 February 2020); *S.* *bicolor* reads were mapped to RTx430 or Sbv3 depending on the experiment (https://phytozome.jgi.doe.gov; last accessed 20 February 2020). SAM alignment file conversion, sorting, and preparation were performed using the Samtools software ver. 1.10 [114]. Counts of the transcripts were determined by featureCounts software with default parameters. Deseq2 ver. 3.10 (R Bioconductor package) was used to identify differentially expressed genes between each pair of samples [115]. Raw counts were normalized by library size and fit to a negative binomial model. Genes with at least a |log2-foldchange| > 0 in expression and Benjamini–Hochberg adjusted *p*-value (q-value) < 0.05 were considered as differentially expressed genes (DEGs). Heatmaps were generated with the pheatmap function (NMF ver. 0.17.6) using -vst values with z-score transformation.

Peroxisomal genes were determined according to the study carried out by [116] in rice and *A. thaliana*. Homologs of peroxisomal genes were determined in other species using orthoFinder (https://github.com/davidemms/OrthoFinder; last accessed 20 February 2020) software [117]. GO terms of counts were annotated by GO annotation files at https://phytozome.jgi.doe.gov, last accessed 20 February 2020. The topGO R Bioconductor package was used for enrichment analysis of DEGs via the Fisher method. Z-scores of GO terms were calculated from the formula using the published procedure [118]. An R script was prepared to examine the results of the GO enrichment analysis [119].

### 2.10. Preparation of Antibodies and Western Blotting

A fragment of *CAT* (GenBank: X94352.1; Supplemental Table S8) corresponding to amino acid residues 96 to 385 was amplified using PCR and cloned into the pDONR207 (Invitrogen) entry vector using the GateWay system. The fragment was verified by sequencing. The fragment was cloned into the pGAT4 destination vector and expressed as a recombinant protein with N-terminal His-Tag in *Escherichia coli* stain Rosetta II (Novagen). 

A fragment of *ATG8* (GenBank#AK457482.1; Supplemental Table S8) corresponding to amino acid residues 1 to 116 was amplified using PCR using forward and reverse primers containing Nhe I and Xho I restriction sites, respectively (Supplemental Table S1). The PCR fragment was cloned in pGEM-T Easy (Promega, Madison, WI, USA) and verified by sequencing. The fragment was released from the pGEM-T Easy by digesting it with Nhe I and Xho I and cloned into expression vector pET28a cut with NdeI and XhoI.

Recombinant CAT and ATG8 were expressed as N-terminal His-Tag fusions in *E. coli* stain Rosetta II (Novagen). Total bacterial protein was extracted using sonication. Recombinant proteins were purified under denaturing conditions in urea buffers on a nickel-nitrilotriacetic acid agarose column (Qiagen). Antibodies were prepared using our established procedure [120,121]. Purified protein was dialyzed against PBS supplemented with 20% glycerol overnight at 28 °C, and protein concentration was adjusted to 1 mg/mL. In total, 50 μg of recombinant CAT or 75 μg of recombinant ATG8 were used for each boost, and a total of 4 boosts occurred over 2 months. Antiserum was collected 10 days after the final boost and tested by immunoblotting.

For the Western blotting with anti-CAT, total protein was extracted from the leaf by crushing the tissue under liquid nitrogen conditions using a mortar and pestle and homogenizing in an extraction buffer containing 50 mM Tris (pH 7.2), 10 mM EDTA, 10 mM Mercaptoethaol, and proteinase inhibitors: 100 μM PMSF, 25 μM Leupeptin, 100 μM Pepstain A, 1 μM E10, and 1 μM MG132. Total protein extract supernatant was mixed 1:1 with 2×SDS-PAGE buffer and boiled for 3 min. Well-watered total protein extract was separated on a 12.5% SDS-PAGE gel and transferred onto a nitrocellulose membrane. The antigen depletion technique was used to test the specificity of the antibody. To prepare the immuno-depleted antibody, recombinant CAT at a final concentration of 10 μg/mL was incubated with primary antibody diluted 1:500 in 2× TBST supplemented with 5% (*w*/*v*) fat-free milk powder at room temperature for 30 min. Then, the nitrocellulose membrane with recombinant CAT was cut into strips and washed for 20 min in the same milk-TBST buffer. One strip was incubated with primary antibody diluted 1:500 in the same buffer, and another strip was incubated with the depleted primary antibody for 1 h at room temperature. Both strips were washed 3 times for 10 min in TBST buffer and incubated with secondary anti-mouse horseradish peroxidase conjugates (Jackson ImmunoResearch, West Grove, PA, USA) diluted to 1:2000 for 35 min. Unbound secondary antibody was washed off in TBST for 10 min each. The signal was imaged and captured using the ECL reagent (GE Healthcare, Boston, MA, USA). 

Total protein was extracted from the leaves of watered and drought-stress wheat plants as described above, mixed 1:1 with 2× SDS-PAGE sample buffer, and boiled for 3 min. Each gel well was loaded with 20 μg/mL of total protein. The extract was run on a 12.5% SDS-PAGE gel and transferred onto a nitrocellulose membrane. The membrane was washed with 2×TBST supplemented with 5% (*w*/*v*) fat-free milk powder for 20 min. The membrane was incubated with primary antibody diluted 1:500 in TBST-milk buffer for 1 hr. The membrane was washed 3 times for 10 min in TBST buffer and incubated with secondary anti-mouse horseradish peroxidase conjugates (Jackson ImmunoResearch, West Grove, PA, USA) diluted to 1:2000 for 35 min. Unbound secondary antibody was washed off in TBST for 10 min each. 

For the Western blotting with anti-ATG8, total protein was extracted from the leaf by crushing the tissue under liquid nitrogen conditions using a mortar and pestle and homogenizing 1:1 with 2× SDS-PAGE buffer and boiled for 5 min. The extracts were separated on a 15% SDS-PAGE gel and transferred onto a nitrocellulose membrane. The antigen depletion technique was used to test the specificity of the antibody exactly as described above. Western blotting was performed as described above, except that the protein extracts were prepared immediately prior to preparing the membranes. 

The membranes were developed by ECL reagent (GE Healthcare), and imaged using a G:BOX Chemi XT4 Gel Imaging System (Syngene, Frederick, MA, USA). The membrane was then washed with agitation 3 times for 10 min with deionized water then total protein was stained with colloidal silver. Total protein values on the colloidal silver-stained membrane and luminescence values on the Western Blotting images were measured using Fiji ImageJ [122]. The luminescence values were normalized by the protein content on the membrane. Statistical differences were analyzed using a Student’s *t*-test. 

To determine the relationship between fluorescent signal and protein abundance, different concentrations of recombinant CAT or ATG-8 protein were prepared by mixing 1:1 with 2× SDS-PAGE sample buffer and boiled for 3 min. Catalase recombinant protein was diluted to final concentrations of 8.6, 17.2, and 34.4 μM, and then 5 μL of each dilution were run on a 12.5% SDS-PAGE gel and transferred onto a nitrocellulose membrane. ATG-8 recombinant protein was diluted to final concentrations of 6.5, 13, and 26 μM, and then 5 μL of each dilution were run on a 15% SDS-PAGE gel and transferred onto a nitrocellulose membrane. Western blotting, image capture and analysis, and colloidal silver staining were performed as described above. 

## 3. Results

### 3.1. Impact of Drought on Root Architecture and Yield

**Figure 1:**
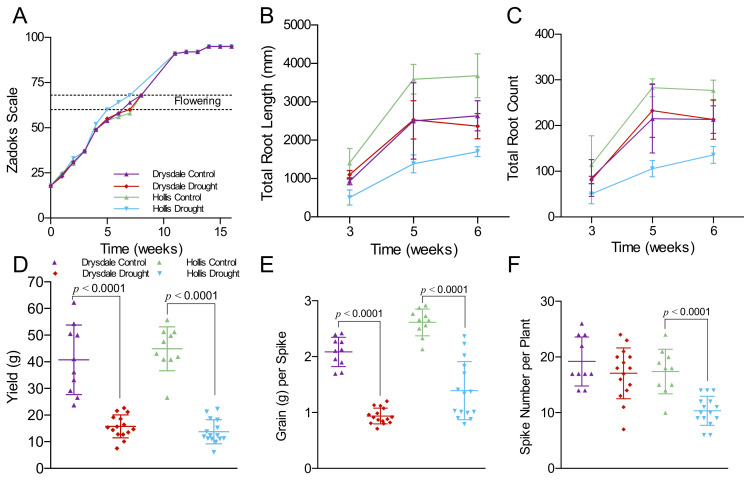
Impact of drought stress on development and yield. (**A**) Comparison of Hollis and Drysdale plant development under normal watering and drought. First and last Zadoks sages corresponding to flowering are denoted by the dotted lines. The average total root length (**B**) and total root count (**C**) of control and drought-stressed Drysdale and Hollis plants. The error bars represent the standard deviation of values from two watered control bins or three drought stress bins each containing five plants. The average yield per plant (**D**), grain weight per spike (**E**), and spike number per plant (**F**) of control and drought-stressed Drysdale and Hollis. Each data point represents average values of the individual plants, *n* = 10 for control and *n* = 15 for drought stress treatment. The statistical significance was analyzed using Student’s *t*-test. Grain yield was significantly reduced (<0.0001) in both genotypes under drought conditions.

Mining soil moisture using longer roots is a known drought avoidance strategy [15,123]. We compared root responses to drought in Drysdale and Hollis. As shallow pots provide poor resolution of root system morphology, we used 55 gallons bins filled with a 90 cm thick layer of soil. Each bin contained two soil moisture probes and one root imaging tube (Supplemental Figure S1A,B). Seedlings were watered normally until the beginning of tillering stage (Zadoks scale 18–19), and then watering was withheld (Figure S1C). The soil WVC was measured at 80 cm (bottom of the bin) and at the 40 cm depths. The moisture declined gradually over the 11 weeks of drought treatment at both depths; however, the depletion rate was faster at the 40 cm level (Supplemental Figure S1D,E). Both genotypes exhibited similar kinetics of soil moisture depletion at each depth (Supplemental Figure S1E). Under normal watering, Hollis and Drysdale flowered at the same time, whereas under drought, Hollis flowered earlier than Drysdale (Figure 1A). Previous measurements in smaller bins under normal watering showed earlier flowering for Hollis [124].

Root systems were analyzed on images taken at different soil moisture values and developmental stages at weeks 3, 5, and 6 (Supplemental Figure S2). At week 3, the VWC values at the top section of the bin were 10–28% (27–32% VWC in the watered controls); at week 5, the VWC values were 5–20%, and at week 6, the values were 2–10%. The decline of the VWC values at the top of the bin was accompanied by the higher values at the bottom of the bean. Thus, a deeper root system in this growth set-up provides access to additional water resources. Additionally, in week 3, plants were in the vegetative growth phase. Week 5 and 6 were right before or immediately after flowering. Later stages were not analyzed as wheat roots cease growth past the flowering stage [124]. Root images were used to measure total root length, total root count, total root volume, total root area, and root diameter. Of these measurements, the total root length and total root count were the most informative because tracking the root thickness in bins with WVC was not possible due lot lower image resolution (Figure 1B,C and Figure S2C–G). Total root length and count were not significantly different in control and drought-stressed Drysdale plants. However, drought stress caused a significant reduction of both root count and length in Hollis at each time point (Figure 1B,C).

We collected three of the following yield parameters at maturity: spike number per plant, grain yield per spike, and total yield per plant (Supplemental Figure S3; Figure 1D–F). Under control conditions, both varieties were similar for the total grain yield (*p* = 0.40) or the number of spikes per plant (*p* = 0.35). Drought caused a significant reduction in grain yield (*p* < 0.0001) in both genotypes (Figure 1D; Supplemental Figure S3), though no significant differences were detected between genotypes. However, the structure of the yield was different: the number of spikes per plant was significantly lower in Hollis than in the Drysdale, whereas the grain weight per spike was greater in Hollis (Figure 1E,F).

### 3.2. Impact of Drought on the Photosynthetic Parameters

**Figure 2:**
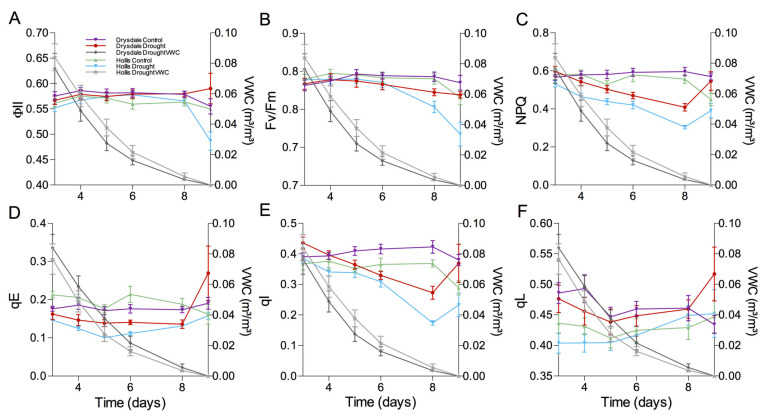
Impact of drought on the photosynthetic parameters. (**A**) Quantum yield of photosystem II photochemistry (ϕ_PSII_). The right Y-axis shows volumetric water content (VWC) at the corresponding time points. (**B**) The quantum efficiency of open photosystem II centers (F_v_/F_m_). (**C**) Non-photochemical quenching (NPQ). (**D**) Energy-dependent quenching (qE). (**E**) Photoinhibitory quenching (qI). (**F**) Open photosystem II centers (qL). The values represent the mean ± SD of 5 biological replicates for both control and drought treatments.

The reduced number of spikes in Hollis plants under drought suggests a decrease in leaf surface area relative to Drysdale. Thus to sustain similar yields, Hollis likely has more efficient photosynthesis per individual leaf. We tested this hypothesis by measuring the chlorophyll fluorescence parameters using a phenomics platform. Plants were grown in 20 cm deep trays to expedite drought. Watering was withheld at the tillering stage (Supplemental Figure S4). Under these experimental conditions, the VWC values declined at the same rate for both varieties over the period of nine days (Figure 2A). The F_v_/F_m_ and ϕ_PSII_ remained unaffected in both varieties until the VWC decreased below 1%. Then, both parameters declined in Hollis but were not significantly affected in Drysdale (Figure 2A,B). The reduction of Fv/Fm indicates that under severe drought, photosystem II in Hollis sustains damages (Figure 2B). Lower ϕ_PSII_ values in Hollis under these conditions (Figure 2B) denote a lower linear electron transport rate. However, the fraction of photosystem II centers in the open (oxidized) state (qL) remained similar in both varieties under drought (Figure 2F). Thus, electron pressure on photosystem II in Hollis and Drysdale was similar.

The NPQ values were significantly lower at all time points in Hollis and, in particular, at VWC values below 1% (Figure 2C). Analysis of two NPQ components, high-energy quenching (qE) and photoinhibitory-dependent quenching (qI), showed similar qE for both genotypes but significantly higher qI values in Drysdale (Figure 2E,F).

**Figure 3:**
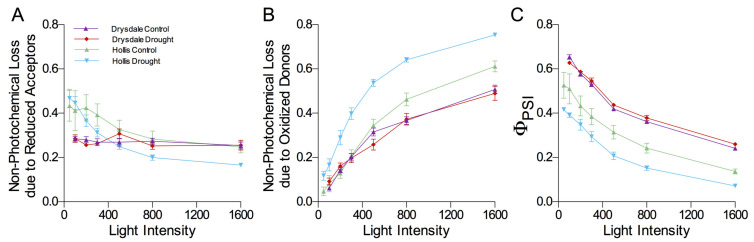
Photochemical analysis of photosystem I. (**A**) Y(NA), non-photochemical loss due to reduced acceptors. (**B**) Y(ND), non-photochemical loss due to oxidized donors. (**C**) Y(I), photoefficiency of photosystem I. The values represent the mean values ±SEM of 5 biological replicates for both control and drought conditions.

To further compare the electron flow in Hollis and Drysdale, we analyzed photosystem I under 1% VWC. The quantum yield of photosystem I photochemistry (ϕ_PSI_) values was significantly lower in Hollis under normal watering and further decreased under drought (Figure 3A). We examined the reason for this difference by measuring the non-photochemical loss due to oxidized electron donors and reduced electron acceptors (Figure 3B,C). According to this analysis, the reduction of ϕ_PSI_ in Hollis under drought is mostly caused by the donor site limitation rather than the acceptor site limitation, whereas ϕ_PSI_ in Drysdale was not affected. This implies that electron transport between photosystems I and II is limited to a greater extent in Hollis than in Drysdale in line with the more reduced primary quinone, QA of PSII (lower qL parameter in Figure 2F).

### 3.3. Activity of the ROS-Scavenging System

**Figure 4:**
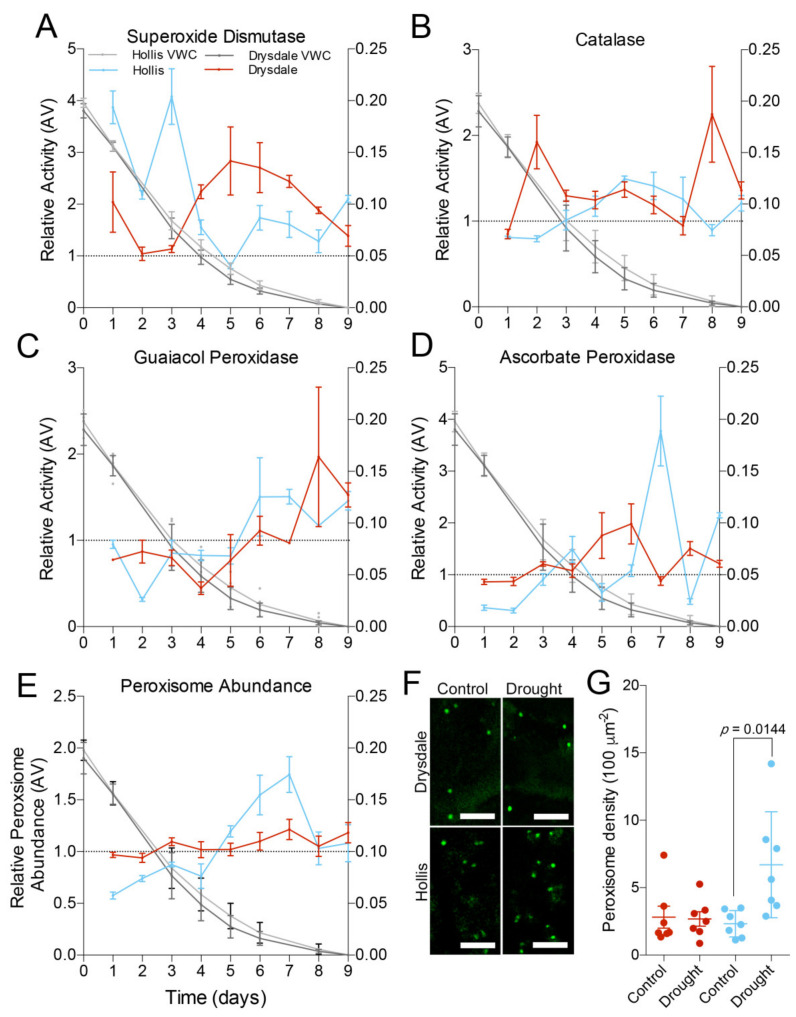
Relative activity of ROS scavenging enzymes during drought stress. The charts show the fold change of the enzyme activity in the drought-stressed samples relative to the control. The right Y-axis shows volumetric water content (VWC) at the corresponding time points. (**A**) Superoxide dismutase. (**B**) Catalase. (**C**) Guaiacol peroxidase. (**D**) Ascorbate peroxidase. (**E**) Peroxisome abundance. The values in A–E represent the mean ± SD of 5 biological replicates for both control and drought. Values above the dashed line indicate up-regulation; values below the line indicate down-regulation. (**F**) Representative images of peroxisomes in leaf epidermis cells of watered and drought-stressed Drysdale and Hollis plants. Scale bar, 5 mm. (**G**) Average density of peroxisomes per 100 mm^2^ of leaf surface in leaf epidermis cells of watered and drought-stressed Drysdale and Hollis plants. Seven cells were measured per each genotype and treatment.

Analysis of ROS scavenging activity in leaf material collected during the drought stress experiment in Figure 2 showed that SOD was more active in Hollis during the beginning of drought and in Drysdale during later stages of drought compared to the well-watered control (Figure 4A). CAT and guaiacol peroxidase were more active during the later stages of drought in both genotypes (Figure 4B,C). The activity of ascorbate peroxidase in Drysdale was higher in the middle of the stress, whereas in Hollis, the activity was higher toward the later drought stages (Figure 4D). These results demonstrate that both genotypes use different components of hydrogen peroxide scavenging throughout drought stress in a dynamic fashion.

### 3.4. Impact of Drought on Peroxisomes

The complexity of the reactions responsible for the maintenance of ROS homeostasis can be assessed using peroxisome abundance [67]. It was shown that hydrogen peroxide and the activity of the ROS scavenging system in leaves correlate with peroxisome abundance [77,78]. We found that the peroxisome abundance in Hollis increased when the VWC decreased below 4% and remained high relative to the control until the last day of treatment. Peroxisomes abundance in Drysdale was not significantly affected through the drought time course (Figure 4E). 

We imaged peroxisomes in leaf epidermis cells using N-BODIPY (Figure 4F) and calculated the density of peroxisomes in 1 μm thick optical sections taken through the cortical cytoplasm (Figure 4G). The average density of peroxisomes was significantly higher in cells from drought-stressed Hollis leaves (*p* = 0.0144), whereas no significant differences were observed between peroxisome density in the control and drought-stressed cells of Drysdale.

**Figure 5:**
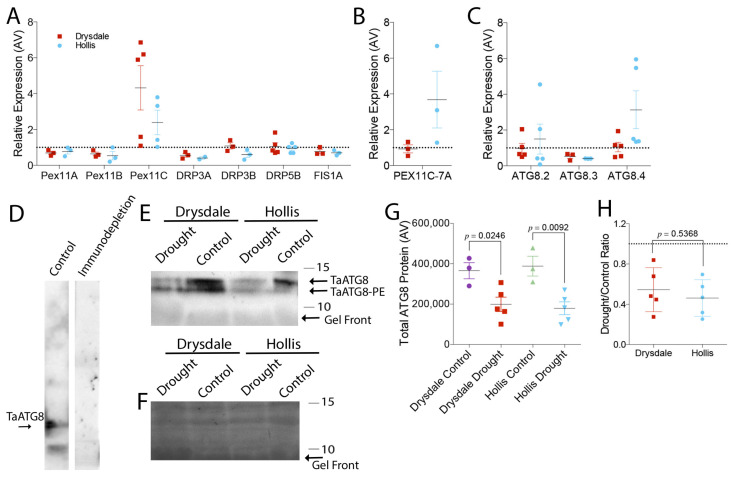
Drought response of peroxisome biogenesis and autophagy markers. (**A**–**C**) Transcription level of peroxisome fission genes (**A**), peroxisome fission gene *PEX11-C* homoeologs from chromosome 7A (**B**) and autophagy flux marker *ATG* (**C**). qRT-PCR transcription levels were normalized to housekeeping gene RNase L inhibitor-like protein. Values above the dashed line indicate up-regulation; values below the line indicate down-regulation. (**D**) Western blotting with ATG8 antibody or following immunodepletion of the antibody with the ATG8 protein. Pre-incubation of the antibody with the antigen abrogates recognition of ATG8 in leaf total protein extract. (**E**) Western blotting with anti-ATG8 of total protein extracts from leaves of control and drought-stressed Drysdale and Hollis plants. Bars and numbers indicate the position and corresponding size of molecular weight markers. (**F**) Colloidal Silver staining of the corresponding Western blotting membrane showing total protein. Bars and numbers indicate the position and corresponding size of molecular weight markers. (**G**) Quantification of ATG8 protein abundance on the Western blotting membranes. *p*-values represent Student *t*-test results of three technical replicates of extracts from three biological replicates (individual plants). (**H**) The ratio of ATG8 protein in extracts from drought-stressed leaves to that in control leaves. *p*-values represent Student *t*-test results of three technical replicates of extracts from three biological replicates (individual plants).

Peroxisome abundance depends on the balance between peroxisome biogenesis and degradation. Peroxisomes proliferate through fission driven by PEX11, FIS1A, DRP3A, DRP3B, and DRP5B. The wheat genome contains three *PEX11* genes, *PEX11A, PEX11B*, and *PEX11C*. We analyzed the transcription of the corresponding genes by qRT-PCR in leaves on day 7 of drought and found that out of seven peroxisome fission genes, only *PEX11C* was upregulated by drought in both genotypes (Figure 5A). Although peroxisome abundance under drought stress was greater in Hollis than in Drysdale, the transcription of *PEX11C* was equally upregulated in both genotypes. The wheat genome has three *PEX11-C* homoeologs on chromosomes 7A, 7D, and 4A [125,126]. qRT-PCR analysis demonstrated that only *PEX11C-7A* was expressed under both control and stress conditions, whereas PCR with primers for the other two homoeologs did not yield a fragment. Transcription of *PEX11C-7A* showed upregulation in Hollis but not in Drysdale in response to drought (Figure 5B). The difference between the generic and homoeologs-specific primers suggests that the generic PEX11C primers could have off-targets.

To find out why peroxisome abundance was different in these genotypes, we assessed the activity of the pexophagy pathway that is responsible for peroxisome degradation. As a type of autophagy, the activity of pexophagy depends on the autophagic flux, which could be determined by the transcription level of *ATG8* [127]. The wheat genome contains 13 putative *ATG8* genes, and six *ATG8* genes were shown to respond to heat and drought stress [128]. Our pilot tests demonstrated that of these six genes, transcription of three genes was upregulated under our drought stress conditions. We found only *ATG8.4* was significantly upregulated in Hollis in response to drought (Figure 5C). The transcriptional analysis was verified by measuring the ATG8 protein abundance under normal and stress conditions using Western blotting with anti-ATG8. The specificity of the antibody was verified using an immuno-depletion assay (Figure 5D), and the intensity of the luminescence was shown to correlate with the protein loading (Supplemental Figure S5A–C). ATG8 abundance under drought decreased in both genotypes relatively to the watered control (Figure 5E–G), though the decrease of ATG8 abundance was somewhat greater in Hollis than in Drysdale (Figure 5H).

Peroxisomes are known to contain ca. 300 different proteins [129,130]. Plausibly, other peroxisome biogenesis genes could be transcriptionally up-regulated in response to drought. We analysed transcription of all annotated peroxisomal genes in response to drought using 19 published RNA-Seq datasets from *Zea mays, Oryza sativa*, *Sorghum bicolor,* and *Arabidopsis thaliana* (Supplemental Table S2). To verify the impact of stress on global gene transcription, we compared the GO term enrichment in the stressed versus control datasets. Genes involved in response to stress, to abiotic stimuli including drought, and stress-induced regulation of gene expression were enriched in the all RNA-Seq datasets for the stress samples relatively to control (Supplemental Table S3). Hence, drought treatment in all experiments induced a stress response. 

**Figure 6:**
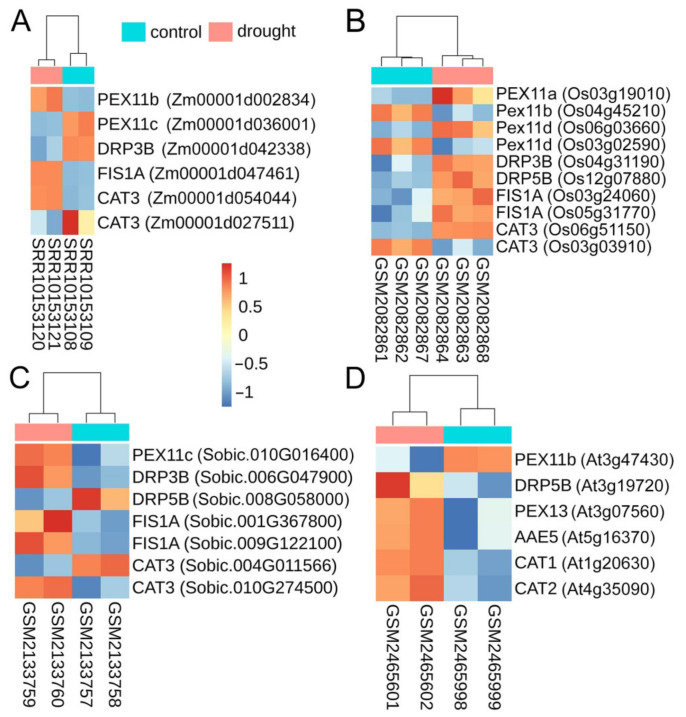
Expression of peroxisome biogenesis genes in response to drought stress. (**A**–**D**), Heatmaps of peroxisome fission genes that are differentially expressed in response to drought stress in *Z. mays* (**A**), *O. sativa* (**B**), *S. bicolor* (**C**), *A. thaliana* (**D**). The figure was generated with the R pheatmap package using VarianceStabilizedTransformation-vst() function built-in DESEq2 package. Vst values were represented based on the z-score transformation. Samples were clustered according to Pearson correlation analysis. Loci name of orthologs that were mapped to peroxisome fission genes are included next to the gene names.

Analysis of the peroxisome genes in these datasets showed that 75 to 120 genes encoding peroxisome proteins were differentially expressed across the species. However, only catalase (*CAT*) and *PEX11* were upregulated in all experiments (Figure 6; Supplemental Tables S4–S7). Interestingly, some members of *CAT* and *PEX11* gene families were down-regulated in response to drought. It means there is functional specialization amongst these gene families under stress. 

**Figure 7:**
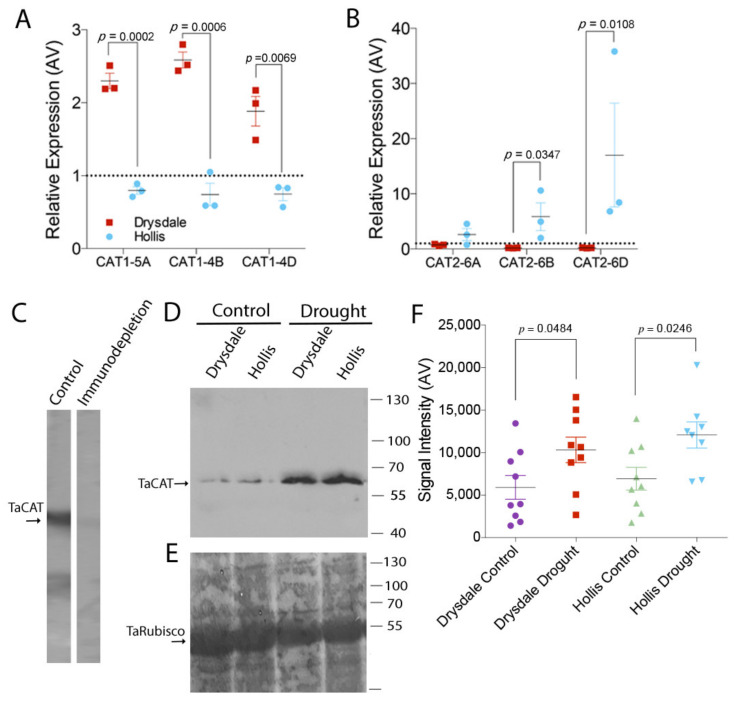
Characterization of Catalase in response to drought stress. (**A**) Transcription of *CAT1* homoeologs in leaves of control and drought-stressed Hollis and Drysdale plants. *p*-values were calculated using Student *t*-test (*n* = 3). (**B**) Transcription of *CAT2* homoeologs in leaves of control and drought-stressed Hollis and Drysdale plants. *p*-values were calculated using Student *t*-test (*n* = 3). Values above the dashed line indicate up-regulation; values below the line indicate down-regulation. (**C**) Western blotting with anti-CAT or following immunodepletion of the antibody with the CAT protein. Pre-incubation of the antibody with catalase abrogates recognition of catalase on the membrane with total protein extract from the leaf of Drysdale plants. (**D**) Western blotting of total protein extracts from leaves of control and drought-stressed Drysdale and Hollis plants. Bars and numbers indicate the position and corresponding size of molecular weight markers. (**E**) Colloidal silver staining of the Western blotting membrane showing total protein in the corresponding extracts. Bars and numbers indicate the position and corresponding size of molecular weight markers. (**F**) Quantification of CAT protein levels on the Western blotting membranes. *p*-values represent Student *t*-test results of three technical replicates of extracts from three biological replicates (individual plants).

We verified outcomes of the RNA-Seq analysis by measuring transcription of *CAT* genes in leaves on the 7th day of drought. *CAT1* is mostly expressed in leaves, *CAT2* is expressed in vascular tissues, and *CAT3* is expressed in reproductive tissues and roots [39,45]. Based on this information, we analysed transcription of *CAT1* and *CAT2. CAT1* homoeologs locate on chromosomes 5A, 4B, and 4D [125,126] and *CAT2* homoeologs locate on chromosomes 6A, 6B, and 6D. Transcription of all three *CAT1* homoeologs under drought was higher in Drysdale than in Hollis, whereas transcription of all three *CAT2* homoeologs was higher under drought in Hollis than in Drysdale with the greatest up-regulation of *CAT2-6D* (Figure 7A,B).

As transcription of *CAT* genes was upregulated under drought in both genotypes, we measured the protein abundance of catalase using Western blotting. The specificity of the anti-catalase antibody was verified using an immuno-depletion assay (Figure 7C), and the intensity of the luminescence was shown to correlate with the protein loading (Supplemental Figure S5D–F). Analysis of the extracts from control and drought-stressed plants demonstrated greater catalase protein abundance under drought relative to the watered control (Figure 7D,E). The average signal was greater in Hollis than in Drysdale, indicating a higher abundance of catalase enzyme in the former genotype (Figure 7F). 

## 4. Discussion

### 4.1. Drought Escape and Avoidance Mechanisms in Hollis and Drysdale

Comparison of two drought-adapted wheat genotypes demonstrated different strategies for drought tolerance. On the developmental level, Hollis relies on the drought escape to a greater extent than Drysdale. First, Hollis flowers one week before Drysdale and matures earlier, as observed in other studies. Second, vegetative growth was reduced in Hollis, as is evident from the reduced size of the root system and lower tiller number, which were greater in Drysdale. A longer time to heading was correlated with increased root proliferation, which could explain the smaller root system in Hollis than in Drysdale [124]. Third, Hollis had higher yield per spike than Drysdale. The latter could be due to greater grain sink strength and capacity, which is determined during the early stages of grain development. In previous studies, Drysdale was found to be a water usage efficient variety [131] and showed that Drysdale had a constitutive advantage in a range of environments for the majority of yield components except for single seed weight [130].

Classical drought escape traits include early flowering and high metabolic rates [13,14]. Earlier transition to flowering may extend the period of grain filling. High metabolic rates facilitate the rapid development and accumulation of photoassimilates which could be used during the grain filling stage. Grain weight is largely determined by starch accumulation [132,133]. Grain sink strength may influence biomass allocation from vegetative growth (roots) to reproductive (grain) [134,135]. Larger grain size was correlated with drought tolerance in rice [136] and wheat [132,133]. 

Root system architecture is another source of drought resistance. Studies in rice [136,137], maize [138,139,140,141], and wheat [123,124,142,143] indicate that longer roots and sharper branching angles can reduce yield losses by improving access to both soil moisture and nutrients. In our study, root length and root number under drought were significantly lower in Hollis than in Drysdale.

The sensitivity of root growth to soil moisture content contributes to drought avoidance by providing greater access to soil moisture. Root growth under low water potential was reported as a drought avoidance trait in wheat [144], *Arabidopsis* [145], soybean [146,147,148], and maize [138,141,149]. Consistent with these reports, Drysdale root growth continues during the period of drought. However, despite a larger root system, the reduction in soil moisture content in containers with Drysdale was similar to that in Hollis. This outcome is consistent with reductions in stomatal conductance in both varieties. Furthermore, reduced stomatal conductance in Drysdale occurs even when roots have access to soil moisture [150]. This means that genotypes with efficient water usage can take advantage of the bigger and deeper root system in deep soils. 

### 4.2. Impact of Drought on Photosynthesis

The impact of drought on photosynthesis was assessed by measuring chlorophyll fluorescence. This approach has been applied to many crops, including barley, bean, and rice. Maintaining photosynthetic efficiency was shown to be an essential drought tolerance mechanism in all plants examined thus far, including rice, maize, wheat, and barley [151,152,153]. In our experiments, both Hollis and Drysdale sustained F_v_/F_m_ and ϕ_PSII_ at the VWC above 1%. The decline in F_v_/F_m_ at severe drought in Hollis provides evidence for photooxidative damage to photosystem II. Consequently, the decrease in linear electron transport rates (indicated by ϕ_PSII_, Figure 2C) could be the consequence of a slower electron injection by PSII into the electron transfer chain [154,155].

Slower electron injection could lead to an excess of energy which can be dissipated through NPQ. Higher NPQ were reported in drought-tolerant tomato [156], maize [157], rice [60,158], and barley [153,159]. Consistent with these findings, we observed similar NPQ values in Drysdale and Hollis at VWC > 1%. However, NPQ values in Drysdale were higher than in Hollis at VWC values below 1%. Analysis of two NPQ components demonstrated a similar fast relaxing component of the NPQ, qE, in both genotypes but lower slower-relaxing components of NPQ, qI, component in Hollis than in Drysdale. A part of the slower-relaxing component could be zeaxanthin-dependent quenching [102]. The observed increase in NPQ in Drysdale relative to Hollis under severe drought is likely to be responsible for reducing the photodamage to photosystem II and maintaining higher F_v_/F_m_. At the same time, F_v_/F_m_ decline in Hollis is indicative of photodamage to photosystem II.

Another reason for reduced F_v_/F_m_ and ϕ_PSII_ in response to drought could be limitations of the electron flux through PSI [104,105]. The ϕ_PSI_ values were significantly lower in Hollis under normal watering and further decreased under drought (Figure 4C). This suggests that drought causes a reduction of the photosystem I donor sites (Figure 4B), ultimately leading to a reduction of electron transport between photosystem II and I. This limitation could be caused by damage to the cytochrome b6f complex by ROS [160,161,162,163] and indicates that Hollis is more likely to experience oxidative stress. 

### 4.3. ROS Scavenging System under Drought

Modification of metabolic processes plays an essential role in drought tolerance. One common change is the accumulation of sucrose, phosphoric acid, and organic acids [164,165]. Drought tolerance correlates with higher transcription of genes involved in fructan metabolism and the accumulation of fructan [164,166]. Another set of metabolic changes focuses on ROS scavenging. Accumulation of ROS in the chloroplast under drought inhibits photosynthesis by causing peroxidation of lipids and oxidative damage to the components of the electron transfer chain [21,33,167]. Plants ameliorate oxidative damage by upregulating the transcription of genes encoding ROS scavenging enzymes *SOD*, *CAT*, and peroxidases [22,43,44,45,46]. The activity of ROS scavengers, including catalase, SOD, ascorbate peroxidase, and glutathione reductase, is upregulated in wheat leaves under drought stress [22,42,43,44,45,46,47,48]. 

As Hollis and Drysdale are drought-tolerant genotypes, both are expected to upregulate ROS scavenging enzymes in response to drought. However, differences in the NPQ, F_v_/F_m_, and ϕ_PSII,_ taken together with different electron flux through PSI, indicate different strategies for handling the excess of ROS production. Consistent with this idea, we found different temporal patterns of ROS scavenging enzyme activity. Hollis mostly relies on ROS scavenging to prevent oxidative damages, whereas Drysdale uses a combination of NPQ and ROS scavenging. 

Drysdale has two peaks for catalase activity at the beginning and the end of the drought. At the same time, Hollis catalase activity is similar to peroxisome abundance peaking towards the end of the drought. SOD activity increases in both genotypes but to a greater degree in Hollis. SOD activity in Drysdale peaks during the middle of the drought. The early activity of SOD could be a result of ROS production during photosynthesis. The reduction of SOD activity during the early stages of drought stress in Hollis could leave the photosystems vulnerable to oxidative damage. This could explain the decline in F_v_/F_m_ and ϕ_PSII_ discussed above. 

Although the overall activity of the ROS scavenging system was consistently elevated under drought stress in Hollis relatively to Drysdale, the activity of individual enzymes varied at different time points after withholding the watering. This variability most likely reflects the non-linear nature of drought responses and some functional redundancy amongst the individual components of the redox system. Hence, measuring the activity of the ROS scavenging system with a limited set of enzymatic assays is prone to misinterpretation. Introducing integrative parameters, such as peroxisome abundance, provides an additional method of assessing the status of the redox system under drought.

### 4.4. Role and Regulation of Peroxisomal Homeostasis in Stress Tolerance

We observed a significant increase in peroxisome abundance under drought only in Hollis. This suggests that both genotypes maintain ROS homeostasis using different mechanisms, one of which is an increase in peroxisome abundance. Peroxisomal proliferation is driven by a set of genes, including *PEX11* [83,85]. Of three wheat *PEX11* genes, transcription of only *PEX11C* was upregulated under drought in both genotypes under our experimental conditions. Analysis of RNA-Seq data from rice, maize, sorghum, and *Arabidopsis* revealed that a member of the *PEX11* gene family was upregulated under drought stress in all species. Interestingly, some members of the *PEX11* gene families were downregulated in response to droughts, such as *PEX11A* and *PEX11B* in this work. In *Arabidopsis,* PEX11A was implicated in the formation of peroxisome extensions known as peroxules under cadmium stress [168], while PEX11B is involved in light-induced peroxisome proliferation [70]. This indicates differences in the peroxisome proliferation process under drought and other stresses.

Dysfunctional peroxisomes are degraded through pexophagy [93,95,169]. The activity of autophagy (the autophagic flux) can be assessed by measuring transcription level *ATG8* [127,170]. One of three *ATG8* measured, *ATG8.4*, was upregulated in both genotypes. The average level of *ATG8.4* transcription was three-fold higher in Hollis than in Drysdale. Another characteristic of the elevated autophagic flux is the degradation of ATG8 protein [171]. We compared the relative abundance of ATG8 in total leaf protein extracts from control and drought-stressed plants. This showed reduced protein content in both genotypes, indicating the activation of autophagy. However, a higher transcription level of *ATG8* accompanied by somewhat lower ATG8 protein abundance in Hollis relative to Drysdale suggests greater autophagic flux under drought stress in Hollis. Autophagic flux was linked to stress tolerance. For example, abiotic stresses activate autophagy, and autophagy-defective mutants are hypersensitive to stresses [172,173]. ROS could modulate or act as a regulator of autophagic responses during abiotic stress [173,174,175].

RNA-seq analysis demonstrated that of all peroxisomal genes, only *PEX11* and *CAT* were upregulated in response to drought in diverse species. Of two *CAT1* and *CAT2* genes, *CAT1* transcription in response to drought was upregulated two-fold in both Drysdale and Hollis, whereas *CAT2* expression was five-fold higher in Drysdale under drought. CAT2 was identified as the enzyme responsible for detoxifying photorespiratory-derived hydrogen peroxide [39]. Overall, *CAT* transcription and CAT protein are good markers of peroxisome abundance. 

Recently, it has been reported that CAT3 can be transnitrosylated and targeted for selective autophagy [176,177]. Ubiquitinated CAT accumulates in a pexophagy adaptor mutant *nbr-1* in *A. thaliana* [178]. NBR1 was established as a cargo receptor for selective autophagy of stress-induced protein aggregates [178] and is implicated in stress responses and tolerance [173,179]. CAT was also shown to co-localize with ATG8 and NBR1 in the electron-dense peroxisomal core in response to cadmium stress [95]. It is plausible that catalase functions as a pexophagy receptor, and a higher protein level of catalase in Hollis is linked to the greater rejuvenation of the peroxisome population in the stressed cells through pexophagy.

Peroxisome abundance and catalase activity patterns overlap in Hollis under drought. This is consistent with the higher transcription of *CAT1*, *CAT2,* and *PEX11C.* Higher catalase activity and the upregulation of *CAT1*, *CAT2*, and *PEX11B* transcription in Drysdale are accompanied by a relatively steady peroxisome abundance. Both varieties had a significant increase in CAT protein. Catalase undergoes multiple post-translational modifications, including carbonylation, S-nitrosylation, and phosphorylation (reviewed by [177]). PEX11 is also a target of post-translational modifications, including phosphorylation, acetylation, and S-nitrosylation [177]. Post-translational modifications could be responsible for fine-tuning the activity of peroxisome fission and degradation processes.

## 5. Conclusions

Drought resistance mechanisms of Drysdale and Hollis share some similarities but also show significant differences. Hollis utilizes drought escape, while Drysdale uses avoidance through adaptive root growth. Drysdale relies more on NPQ mechanisms for maintaining high photosynthetic rates under drought. Dynamic responses in ROS scavengers were found in both varieties. Hollis exploits peroxisome abundance and autophagic flux to combat stress-derived ROS and oxidative damages more efficiently than Drysdale. Peroxisomes appear to be an essential component of drought adaptation. The identification of genetic markers of peroxisome proliferation and autophagic flux is essential for breeding wheat with greater stress resiliency. 

## Data Availability

All data and materials are available on request.

## Supplementary Materials

The following supporting information can be downloaded at: https://www.mdpi.com/article/10.3390/cells11111765/s1, Figure S1. Experimental setup for measuring the impact of drought on root growth and yield; Figure S2. Analysis of root growth response to drought stress; Figure S3. Images of bins taken prior to harvest; Figure S4. Representative images of Hollis and Drysdale plants taken at different stages of drought stress; Figure S5. Correlation between protein loading and Western blotting signal for wheat ATG-8 and CAT antibodies; Table S1. Sequences of primers used in this work; Table S2. NCBI SRA RNA-Seq datasets used in the study; Table S3. Enrichment of gene ontology terms related to stress response in the RNA-Seq datasets [56,180,181,182,183,184,185,186]; Table S4; Impact of drought on transcription of peroxisome biogenesis genes in *A. thaliana*; Table S5. Impact of drought on transcription of peroxisome biogenesis genes in *Z. mays*; Table S6. Impact of drought on transcription of peroxisome biogenesis genes in *S. bicolor*; Table S7. Impact of drought on transcription of peroxisome biogenesis genes in *O. sativa*. Table S8. Nucleic acid sequences of the genes used in the study.
