# Supplementary material for: Drought Tolerance Strategies and Autophagy in Resilient Wheat Genotypes
Source: Cells. 2022 May 27;11(11):1765. doi: 10.3390/cells11111765 (PMC9179661; doi:10.3390/cells11111765)
Supplement: Supplementary file 1 [file cells-11-01765-s001.zip › cells-1714556-supplementary.pdf]

Supporting Information

Autophagy and ROS homeostasis compensate for drought-induced damages to photosynthetic apparatus

Kathleen Hickey[1], Magnus Wood[1], Tom Sexton[2], Yunus Sahin[1], Taras Nazarov[1],Jessica Fisher[1], Karen A. Sanguinet[3], Asaph Cousins[2], Helmut Kirchhoff[1]*, Andrei Smertenko[1]*

[1]Institute of Biological Chemistry, Washington State University, Pullman, WA, USA
[2]School of Biological Sciences, Washington State University, Pullman, WA, USA
[3]Department of Crop and Soil Sciences, Washington State University, Pullman, WA, USA

Supplemental Table S1. Sequences of primers used in this work.

Gene Name	Type	Forward Primer	Reverse Primer
Catalase	Gateway cloning	GGGGACAAGTTTGTACAAAAAAGCAGG<br>CTTCGAACGCATACCTGAACGTGTTGTT<br>C	GGGGACCACTTTGTACAAGAAAGCTGG<br>GTCTCACACATTTTGGAGCATTGACTG
Catalase-1	qPCR	CACCTGGTGGAGAAGATCGC	TCACCTCGAAGAAGCCCTTG
Catalase-2	qPCR	AACATGCAGGAGAACTGGAGAG	CATGTGCCTGTAGTTGAGTGGA
ATG8	Traditional Cloning	ACATATGAAATCCTTCAAGAAGGAATTC<br>AC	ACTCGAGTCACCCAAATGTCTTCTCGCT<br>GC
Rnase L Inhibitor Like Protein (RLI)	qPCR	CGATTCAGAGCAGCGTATTGTTG	AGTTGGTCGGGTCTCTTCTAAATG
PEX11-A	qPCR	CGCTAGGGGACGTGACTAA	CAGCGCCGACAGCAATC
PEX11-B	qPCR	CAACCCGTTCTGCAACCAC	TTCCTATACCACCCAGCCCA
PEXX11-C	qPCR	GAAGAACGCGATGCTGTCAA	TAAAAGGCAATCCTGCCAAG
DRP-3A	qPCR	GACCTGCGGAGACAATGATAAC	GTTGGTCCTCTCGAAGATAGA
DRP-3B	qPCR	TGGACGAGATACCGCTTGAA	CACTGAAAGGTTGTTGCTGC
FIS-1A	qPCR	TCCAAGCAGACTGATGATGTG	TGGGCTGGTGGTTTTATCAAGA
ATG-8.2	qPCR	TCGATCGCAGGTTGGAGATG	TTGATCCTCTTCCGCACCAC
ATG-8.3	qPCR	CTGGAAAGGAGGCAAGCTGA	TTGATCCTCTTCCGCACCAC
ATG-8.4	qPCR	TCTCGCCTCCTCCCTTTACT	TTGAACGAGCTCTTCGCCAT
CAT1-5A	qPCR	GGCCGAATTACCTGCTGCT	AAGTAGTCGACCTCCTCGTCG
CAT1-4B	qPCR	GGACTATGAGGAGCGGTTCG	GTTGTCGATGTTGCGGTTC
CAT1-4D	qPCR	CAGGCTGACAAGTCTCTCGG	TTGGTACGTAATCCTCGCCG
CAT2-6A	qPCR	TCAAGAGAGGAAGGATGGAT	GTAGTCCTCCAGCAAGATCG
CAT2-6B	qPCR	CCAGCTTAGCTGAGCTACTT	CTCGTTGTCGTTCCACACG
CAT2-6D	qPCR	GGATGGATCGATCTGCCAT	AGCAGCACAGTATGTAATCGA
PEX11-7A	qPCR	CCAGCTTACTACACAGGCA	TGTGACGGTGTCCAATGAG
PEX11-4A	qPCR	TAGCCAGCTTACACACACAA	CTCTGACGGTGTCCACTGA
PEX11-7D	qPCR	CCATCCAATAGCCTGGTT	ATGCGAGCCATCACCATT

Supplemental Table S2. NCBI SRA RNA-Seq datasets used in the study.

GEO Accession No.	Stress	Species	Genotype	Tissue	Stress duration	Repli-cates	Study location	Reference
**GSE134945**	Drought	*A. thaliana*	Columbia (WT)	Leaf	7d,14d, 21 d	3	Growth chamber	https://doi.org/10.1186/s12864-019-6350-5
**GSE93979**	Drought	*A. thaliana*	Columbia (WT)	Leaf	21 d	3	Growth chamber	https://doi.org/10.1093/jxb/erx157
**GSE78972**	Drought (long day)	*Oryza Sativa*	Nipponbare (WT)	Leaf	8 weeks	3	Growth chamber	https://doi.org/10.1111/pce.12760
**GSE78972**	Drought (short day)	*Oryza Sativa*	Nipponbare (WT)	Leaf	6 weeks	3	Growth chamber	https://doi.org/10.1111/pce.12760
**GSE65022**	Drought	*Oryza Sativa*	Nipponbare (WT)	Leaf	10 d	2	No info	No publication
**GSE57950**	Drought	*Oryza indica*	Huanghuazhan (P28) (Tolerant)	Leaf	1-3 d	2	Greenhouse	https://doi.org/10.1186/1471-2164-15-1026
**GSE57950**	Drought	*Oryza indica*	Huanghuazhan (H471) (Tolerant)	Leaf	1-3 d	2	Greenhouse	https://doi.org/10.1186/1471-2164-15-1026
**GSE57950**	Drought	*Oryza indica*	Huanghuazhan (HHZ) (Susceptible)	Leaf	1-3 d	2	Greenhouse	https://doi.org/10.1186/1471-2164-15-1026
**GSE132113**	Drought	*Zea mays*	B104	Leaf (V9 stage)	5 d	2	Field experiments	https://doi.org/10.1186/s12870-019-1941-5
**SRP106756 (PRJNA385354)**	Drought	*Zea mays*	M54 and 753F	Leaf	1d, 2d, 3d	1	No info	No publication
**SRP132035 (PRJNA432650)**	Drought	*Zea mays*	No info	Leaf	No info	4	No info	No publication
**GSE48507**	Drought (Dehydration stress)	*Zea mays*	B73	Seedling	6h and 66 h	2	Green house	https://doi.org/10.1186/1471-2229-14-141
**GSE137780**	Drought	*Zea mays*	B104	Leaf	No info	2	No info	No publication
**GSE137780**	Drought	*Zea mays*	DH4866	Leaf	No info	2	No info	No publication
**GSE137780**	Drought	*Zea mays*	W22	Leaf	No info	2	No info	No publication
**GSE128441**	Drought	*Sorghum Bicolor*	RTX430	Leaf	3 week	3	Field experiment	https://doi.org/10.1073/pnas.1907500116
**GSE128441**	Drought	*Sorghum Bicolor*	RTX430	Leaf	6 week	3	Field experiment	https://doi.org/10.1073/pnas.1907500116
**GSE80699**	Drought	*Sorghum Bicolor*	IS20351 (Sensitive)	Leaf	8 days	3	Controlled Field experiment	https://doi.org/10.1186/s12870-016-0800-x
**GSE80699**	Drought	*Sorghum Bicolor*	IS20330 (Tolerant)	Leaf	8 days	3	Controlled Field experiment	https://doi.org/10.1186/s12870-016-0800-x

Supplemental Table S3. Enrichment of gene ontology terms related to stress response in the RNA-Seq datasets.

Dataset	Species	Term	GO_ID	Gene_ratio	Fold change	Q-value
**GSE137780_DH4866**	Maize	Defense response	GO:0006952	20/51	-6.86/4.68	0.022
		negative regulation of gene expression	GO:0045814	3/6	-2.19/-0.89	0.046
		regulation of cyclin-dependent protein s...	GO:0000079	4/18	-2.02/2.39	0.008
**GSE137780_B104**	Maize	Response to oxidative stress	GO:0006979	31/185	-4.68/3.74	0.032
		Response to abiotic stimulus	GO:0009628	10/27	-2.88/6.51	0.024
		regulation of cyclin-dependent protein kinase	GO:1904029	4/18	-2.09/ -0.67	0.001
**GSE137780_W22**	Maize	regulation of cellular component organization	GO:0051128	15/56	-2.17/2.07	0.035
		regulation of biosynthetic process	GO:0009889	413/1320	-8.30/7.35	0.035
		regulation of cyclin-dependent protein synthesis	GO:0000079	12/18	-1.13/2.02	0.001
		regulation of actin polymerization or depolymerization	GO:0008064	11/26	-2.17/2.07	0.000
		regulation of cellular component organization	GO:0051128	13/56	-0.70/4.25	0.000
**GSE78972S**	Rice	response to external stimulus	GO:0009605	207/494	-6.65/8.27	0.000
		regulation of gene expression, epigenetics	GO:0040029	65/128	3.28/5.08	0.000
		Regulation of anatomical structure size	GO:0090066	169/465	-12.15/5.71	0.000
		abscission	GO:0009838	9/29	-2.93/0.56	0.000
**GSE78972L**	Rice	response to abiotic stimulus	GO:0009628	1118/2194	-9.52/9.71	0.000
		response to extracellular stimulus	GO:0009991	105/304	-9.15/9.71	0.001
		regulation of biological process	GO:0050789	710/1588	-8.13/8.73	0.000
		regulation of cell size	GO:0008361	179/465	-10.98/8.52	0.000
		regulation of gene expression, epigeneti...	GO:0040029	64/128	-2.48/3.31	0.000
		anatomical structure morphogenesis	GO:0009653	343/886	-10.98/8.52	0.000
		abscission	GO:0009838	9/29	-2.60/3.27	0.000
**GSE65022**	Rice	response to extracellular stimulus	GO:0009991	79/304	-6.71/9.40	0.000
		regulation of cell size	GO:0008361	122/465	-6.71/7.11	0.000
		regulation of cellular process	GO:0050794	524/1464	-8.69/8.72	0.005
		peroxisome	GO:0005777	100/191	-5.63/8.48	0.000
**GSE57950P**	Rice	response to stress	GO:0006950	1106 /3618	-10.35/9.82	0.036
		response to abiotic stimulus	GO:0009628	736/2194	-10.35/9.82	0.00
		regulation of anatomical structure size	GO:0090066	99/465	-7.03/4.58	0.013
		regulation of biological process	GO:0050789	498/1588	-8.8/6.00	0.000

		regulation of gene expression	GO:0010468	34/128	-2.68/1.70	0.000
**GSE57950HZ**	Rice	regulation of anatomical structure size	GO:0090066	127/465	-9.66/4.45	0.000
		regulation of cell size	GO:0008361	127/465	-9.66/4.45	0.001
		regulation of gene expression	GO:0010468	46/128	-4.16/1.72	0.000
		Signal transduction	GO:0007165	533/1464	-9.75/11.15	0.001
**GSE57950H**	Rice	response to stress	GO:0006950	1198/3618	-10.63/10.41	0.000
		response to external stimulus	GO:0009605	132/494	-9.56/4.61	0.000
		regulation of cellular process	GO:0050794	501/1464	-9.09/7.22	0.018
		peroxisome	GO:0005777	99/191	-8.73 / 3.01	0.000
**GSE128441W3**	Sorghum	regulation of metabolic process	GO:0019222	104/1170	-2.69/7.58	0.003
**GSE128441W6**	Sorghum	regulation of cellular process	GO:0050794	106/1586	-2.75/3.44	0.027
		regulation of transcription	GO:0006355	65/1077	-2.75/3.44	0.000
**GSE80699_IS20351**	Sorghum	integral component of peroxisomal membrane	GO:0005779	3/7	0.86/2.01	0.000
		regulation of transcription	GO:0006355	107/996	-8.37/7.47	0.012
**GSE80699_IS22330**	Sorghum	cellular response to stress	GO:0033554	13/141	-4.67/-4.49	0.000
**GSE93979**	Arabidopsis	response to stress	GO:0006950	577/3887	-9.67/7.98	0.000
		regulation of response to osmotic stress	GO:0047484	6/37	-1.01/4.19	0.010
		regulation of response to external stimulus	GO:0032101	20/102	-0.86/2.18	0.000
		response to jasmonic acid	GO:0009753	42/208	-4.78/4.22	0.000
**GSE101488**	Arabidopsis	response to salt stress	GO:0009651	34/476	-2.13/5.18	0.000
		cellular response to endogenous stimulus	GO:0071495	60/1050	-4.19/4.87	0.000
**GSE108610**	Arabidopsis	response to oxidative stress	GO:0006979	93/459	-5.36/3.37	0.000
**GSE134945**	Arabidopsis	auxin-activated signaling pathway	GO:0009734	13/208	-2.82/1.02	0.001

Supplemental Table S4. Impact of drought on transcription of peroxisome biogenesis genes in *A. thaliana*.

Gene	mRNA-Seq Datasets							
	GSE93979	GSE134945	GSE108610	GSE101488	GSE93979	GSE134945	GSE108610	GSE101488
	Drought				Control			
PEX11b	up	-	-	-	-	-	-	-
DRP5B	up	-	-	-	-	-	-	-
PEX13	up	-	-	-	-	-	-	-
CAT1	up	-	-	-	-	-	-	-
CAT2	-	-	-	-	up	-	-	-
PEX19B	-	-	-	up	-	-	-	-

Supplemental Table S 5. Impact of drought on transcription of peroxisome biogenesis genes in *Z. mays*. Genes marked in red show inconsistent expression pattern between control and drought-stress mRNA-Seq datasets.

Gene	mRNA-Seq Datasets											
	GSE132113	GSE48507	GSE137780_B104	GSE137780_DH4866	GSE137780_W22	SRP132035	GSE132113	GSE48507	GSE137780_B104	GSE137780_DH4866	GSE137780_W22	SRP132035
	Drought						Control					
PEX11C	up	up	up	-	-	-	-	-	-	-	up	-
PEX11B	-	-	-	-	up	up	-	-	-	-	-	-
PEX11D	-	up	-	-	-	-	-	-	-	up		up
CAT3 (zm00001d014848)	up	up	up	-	-	-	-	-	-	-		-
CAT3 (zm00001d027511)	-	-	-	-	-	up	up	up	up	up	up	-
CAT3 (zm00001d054044)	-	-	-	-	up	-	up	up	up	-	-	up
FIS1A (zm00001d 047461)	-	-	-	-	up	-	-	-	-	-	-	-
DRP3B	-	-	-	-	-	-	-	-	-	up	up	up
PEX1	-	-	-	-	-	-	-	-	-	-	up	up
PEX2	-	-	-	-	-	-	-	-	-	-	up	-
PEX3B	-	-	-	-	-	-	-	-	-	-	-	up
PEX4	up	-	-	up	up	-	-	-	up	-	-	-
PEX5	-	-	-	-	-	-	-	-	-	-	-	up
PEX6 (zm00001d026313)	-	up	-	-	-		-	-	-	up	up	-
PEX6 (zm00001d002462)	-	up	-	-	-		-	-	-	up	-	-
PEX10	-	-	-	-	-	-	-	-	-	-	-	up
PEX12	-	-	-	-	-	-	-	-	-	up	up	up
PEX13	up	-	up	-	up	-	-	-	-	up	-	-
PEX16	-	-	-	-	-	-	-	-	-	-	up	up
PEX19A	-	-	-	up	up	-	-	-	-	-	-	up
PEX22(zm00001d026361)	-	-	-	-	-	up	-	-	-	-	-	-
PEX22(zm00001001849)	-	-	-	-		-	-	-	-	-	-	up

Supplemental Table S6. Impact of drought on transcription of peroxisome biogenesis genes in *S. bicolor*.

Gene	mRNA-Seq Datasets							
	SE12844 (w3)	SE12844 (w6)	IS20351	IS20330	SE12844 (w3)	SE12844 (w6)	IS20351	IS20330
	DROUGHT				CONTROL			
DRP3B	UP	-	-	UP	-	-	-	-
DRP5B	-	-	-	-	-	-	-	UP
PEX11B	-	-	UP	-	-	-	-	-
PEX11C	-	-	UP	UP	-	-	-	-
PEX11D	-	-	-	-	UP	-	-	-
FIS1A Sobic.001G367800)	-	-	-	UP	-	-	-	-
FIS1A Sobic.009G122100)	-	-	-	UP	-	-	-	-
CAT3 (Sobic.010G274500)	-	-	UP	UP	-	-	-	-
CAT3 (Sobic.004G011566)	-	-	-	UP	-	-	-	-
PEX4	-	-	-	UP	-	-	-	-
PEX5	-	-	UP	-	-	-	-	-
PEX10	-	-	UP	-	-	-	-	-
PEX14	-	-	-	UP	-	-	-	-
PEX22		UP	UP	UP				

Supplemental Table S7. Impact of drought on transcription of peroxisome biogenesis genes in *O. sativa*. Genes marked in red show inconsistent expression pattern between control and drought-stress mRNA-Seq datasets.

Gene	mRNA-Seq Datasets											
	GSE78972 (long)	GSE78972 (short)	GSE65022	GSE57950 (P28)	GSE57950 (H471)	GSE57950 (HHZ)	GSE78972 (long)	GSE78972 (short)	GSE65022	GSE57950 (P28)	GSE57950 (H471)	GSE57950 (HHZ)
	Drought						Control					
PEX11C	up	up	up	up	up	up	-	-	-	-	-	-
PEX11A(LOC_OS03G19010)	up	up	up	-	up	-	-	-	-	-	-	-
PEX11B	-	-	-	-	-	-	up	up	up	up	up	up
PEX11D	-	-	-	-	-	-	up	up	up	-	-	-
DRP3B	up	up	-	-	-	-	-	-	-	-	-	-
DRP5B	up	up	-	up	up	up	-	-	-	-	-	-
CAT3 (LOC_os06g51150)	up	up	up	up	up	up	-	-	-	-	-	-
CAT3(LOC_os03g03910)	-	-	-	-	-	-	up	up	up	up	up	up
CAT3(LOC_os02g02400)	-	-	-	-	up	up	-	up	up	-	-	-
FIS1A (LOC_os03g24060)	up	up	-	-	-	-	-	-	-	-	-	up
FISA (LOC_os05g31770)	-	up	-	-	-	-	-	-	-	up	up	up
PEX1	up	-	-	-	-	-	-	up	-	-	-	-
PEX2	-	-	-	-	-	-	up	-	-	up	-	up
PEX3B	-	-	-	-	up	up	-	-	-	-	-	-
PEX4	-	-	-	-	-	-	up	up	-	up	-	up
PEX5	-	-	-	-	up	up	-	-	-	-	-	-
PEX6	-	-	-	-	-	-	up	up	-	-	-	-
PEX7	-	up	up	-	-	-	-	-	-	up	-	-
PEX10	-	-	-	-	-	-	up	up	-	-	-	-
PEX12	-	-	-	-	-	-	up	up	-	-	-	-
PEX13	-	-	up	-	-	-	up	up	-	up	-	up
PEX14	-	-	-	-	-	-	-	up	-	-	-	-
PEX16	up	-	-	-	up	up	-	-	-	-	-	-
PEX19A	-	-	-	-	-	-	-	up	-	-	-	-
PEX22 (LOC_os04g53690)	-	-	-	-		-	up	-	-	-	-	-

Supplemental Table S8. Nucleic acid sequences of the genes used in the study.

Gene	NCBI Accession Number	Nucleic Acid Sequence
ATG8	AK457482.1	ATGAAATCCTTCAAGAAGGAATTCACCCTGGAGGAGAGGGCGAATGAGTC<br>GGCCGCCATGATCGCCAAGTACCCCGGCAGGATCCCCGTGATTGTTGAA<br>AGGTTTTCAAGGAGTAATCTTCCAGAAATGGAGAAGAGGAAGTACCTGGT<br>TCCTTGTGACATGCCAGTTGGGCAGTTCATTTTCATCCTGCGCTCCAGGTT<br>ACATCTGTCTCCAGGGACGGCGCTTTTCGTGTTTGTGCGCGACACCTTGC<br>CCCAGACCGCTAACCTGATGGGTAGCGTGTATGATTCGTACAAAGATAAG<br>CAGGATGGCTTCCTCTACATGTGTTACAGCAGCGAGAAGACATTTGGGTG<br>A
Catalase	X94352.1	ATGGATCCCTACAAGCACCGGCCCACGAGCGGGGCCAACTCCGCCTACT<br>GGACCACCAACTCCGGCGCCCCCGTCTGGAACAACAACAACGCCCTCAC<br>CGTCGGACACCGAGGACCTATCCTCCTTGAGGATTACCATCTGATTGAAA<br>AGCTTGCACAATTTGACCGGGAACGCATACCTGAACGTGTTGTTCATGCA<br>CGGGGAGCCAGTGCAAAGGGGTTCTTTGAGGTGACTCATGATGTTTCTCA<br>GCTCACATGTGCTGACTTTCTCCGGGCTCCTGGGGTTCAGACCCCGGTTA<br>TTGTCCGGTTCTCTACCGTTGTGCATGAGCGTGGAAGCCCTGAGACCCTC<br>AGGGATCCACGTGGTTTTGCAGTGAAGTTCTACACCAGAGAGGGTAACTT<br>TGACCTTGTTGGGAACAATATGCCTGTGTTTTTTATCCGAGATGGGATGAA<br>GTTCCCTGACATGGTCCATGCTTTCAAGCCAAGTTCGAAGACCAACATGC<br>AGGAGAACTGGAGAGTAGTTGACTTCTTTTCGCACCACCCGGAGAGTCTG<br>CACATGTTCACCTTCCTATTTGACGATGTTGGCATTCCACTCAACTACAGG<br>CACATGGACGGTTTTGGTGTCAACACCTACACCTTAATCAGCAGGGATGG<br>AAAGGCTCACCTGGTTAAGTTCCATTGGAAACCTACATGTGGTGTGAAGT<br>GCCTCTTGGATGATGAAGCTGTTACTGTTGGAGGCACCTGCCACACCCAT<br>GCCACAAAGGACTTGACTGATTCTATTGCAGCTGGGAATTACCCAGAATG<br>GAAGCTTTTCATCCAGACCATTGATGCTGATCACGAGGATAGATTTGACTT<br>TGACCCTCTTGATGTCACCAAGACCTGGCCAGAGGACATCATCCCACTGC<br>AACCAGTTGGACGGATGGTACTGAACAAGAACATTGACAATTTCTTCGCG<br>GAAAATGAACAGCTTGCTTTCTGCCCAGCAGTCACTGTCCCTGGAATCCA<br>CTACTCTGATGATAAGCTGCTCCAGACAAGGATCTTCTCATATGCTGATAC<br>CCAAAGGCACCGTCTTGGTCCAAACTACTTGATGCTCCCCGTGAATGCCC<br>CGAAATGTGCTCACCACAACAACCATCATGATGGCTTAATGAATTTCATTC<br>ACAGGGATGAGGAGGTGAACTACTTCCCTTCAAGGGTTGATCCTACTCGT<br>CATGCTGAAAAGGACCCTATGCCTCCGCGTGTTCTATCTGGCTGCCGGGA<br>GAAGTGCATTATCGACAAGGAGAACAATTTCAAGCAGGCTGGTGAGAGAT<br>ATCGGTCCTTCGACCCTGCCAGGCAAGACCGTTTCCTCCAGCGGTGGGTT<br>GATGCGCTCACGGATGCTCGTGTCACCCATGA
PEX11C	AK456439	ATGGCCTCGCTGGACACCGTCAGAGGAGATCTTGGCCTGGTTGTTTTGTA<br>CCTAAGCAAGGCTGAGGCAAGAGATAAGATCTGTAGAGCTATACAATATGGA<br>TCCAAGTTCCTGAGCAACGGACAACCAGGACCTGCACAGAATGTCGACAA<br>ATCAACTAGTCTAGCTCGGAAAGTTTTCCGACTGTTTAAGTTTGTTAATGAT<br>CTCCAAGCTCTGATTAGCCCCCCTGCCAAAGGAACTCCACTTCCGCTGATC<br>TTACTTGGAAAGTCGAAGAACGCGATGCTGTCAACTTTCCTCTTTCTGGAC<br>CAAATTGTTTGGGCTGGGAGAACAGGAGTATACAAGAACAAGGAGCGAGC<br>AGAGTTTCTTGGCAGGATAGCATTTTATTGCTTTCTCGGATCCAATACCTGT<br>ACTACTATCATCGAGCTAGCCGAGCTTCAGCGGCTTTCCAAATCGATGAAG<br>AAGTTAGAGAAGGACCTCAAGCACCAAGAGCTGTACAAGAACGAGCAGTA<br>TCGGATGAAGCTGAAGAAGTCCAACGAGAGGCTGCTCGCCCTCATCAAAT<br>CGAGCCTCGACATAGTCGTCGCCGTGGGGCTGCTGCAACTGGCACCAAA<br>AAAGGTCACTCCTCGCGTCACGGGGGCGTTTGGGTTCGCTAGCTCGCTCA<br>TCGCCTGTTACCAGTTGCTTCCAGCCCCAGCCAAATCCAAGTGATCTGCCT<br>GCCCACCTCGGGCCAGGCAGCTGGTGCATCGATTGGTGAAACGGCGGTT<br>GCTGTCGCCCCTTGTTGTCGCTGATACTGATAAGAAATAAATCTGGACCGA<br>ACTGTCCGTCCCCGTAGGATATAACCGAGGATTATTGCTGTGCATGCTGGC<br>ATTGCCGAGGGCCATCTCATAGCCTGGTTGATCTGCCTTGCCTTAGACTTG<br>ATCATGTAGCACGCTTGTATTTGTATGGTCCGGTGTGCCACTATCATACTAC<br>AACTGTTGCGAAATAATTAGAAATTTCATACTAGAATGGTGATGGCTCGCGT

		CTAGATTTATCTTGACGTTGTGCCGCGACGATGA
PEX11C-7A	AK456439.1	ATGGCCTCATTGGACACCGTCACAGGAGATCTTGGCCTGGTTGTTTTGTAC<br>CTAAGCAAGGCTGAGGCAAGAGATAAGATCTGCAGAGCTATACAATATGGA<br>TCCAAGTTCCTGAGCAACGGACAACCAGGACCTGCACAGAATGTCGACAA<br>ATCAACTAGTCTTGCTCGGAAAGTTTTCCGGCTGTTTAAGTTTGTTAATGAT<br>CTCCAAGCTCTGATTAGCCCCCCTGCCAAAGGAACTCCACTTCCGCTGATC<br>TTACTTGGAAAGTCGAAGAACGCGATGCTGTCAACTTTCCTCTTTCTGGAC<br>CAAATTGTTTGGGCTGGGAGAACAGGAGTATACAAGAACAAGGAGCGAGC<br>AGAATTCCTTGGCAGGATTGCCTTTTATTGCTTTCTTGGATCTAATACATGTA<br>CTACCATCATCGAGCTAGCGGAGCTTCAGCGGCTTTCCAAATCGATGAAGA<br>AGTTAGAGAAGGACCTCAAGCACCAAGAGCTGTACAAGAACGAGCAGTAT<br>CGGATGAAGCTGAAGAAGTCCAATGAGAGGCTGCTTGCTCTCATCAAATCG<br>AGCCTTGACATAGTTGTCGCAGTGGGGCTGCTGCAACTGGCCCCGAAAAA<br>GGTCACTCCTCGCGTCACTGGGGCCTTTGGGTTCGCTAGCTCGCTCATCG<br>CCTGTTACCAGTTGCTTCCAGCCCCAGCCAAATCCAAGTGA
PEX11C-7D	XM_037603080.1	ATGGCCTCATTGGACACCGTCAGAGGAGATCTTGGCCTGGTTGTTTTGTAC<br>CTAAGCAAGGCTGAGGCAAGAGATAAGATCTGCAGAGCTATACAATATGGA<br>TCCAAGTTCCTGAGCAACGGACAACCAGGACCTGCACAGAATGTCGACAA<br>ATCAACTAGTCTTGCTCGGAAAGTTTTCCGGCTGTTTAAGTTTGTTAATGAT<br>CTCCAAGCTCTGATTAGCCCCCCTGCCAAAGGAACTCCACTTCCGCTGATC<br>TTACTTGGAAAGTCGAAGAACGCGATGCTGTCAACTTTCCTCTTTCTGGAC<br>CAAATTGTTTGGGCTGGGAGAACAGGAGTATACAAGAACAAGGAGCGAGC<br>AGAATTCCTTGGCAGGATTGCCTTTTATTGCTTTCTTGGATCTAATACATGTA<br>CTACCATCATCGAGCTAGCGGAGCTTCAGCGGCTTTCCAAATCGATGAAGA<br>AGTTAGAGAAGGACCTCAAGCACCAAGAGCTGTACAAGAACGAGCAGTAT<br>CGGATGAAGCTGAAGAAGTCCAATGAGAGGCTGCTTGCTCTCATCAAATCG<br>AGCCTTGACATAGTTGTCGCAGTGGGGCTGCTGCAACTGGCCCCGAAAAA<br>GGTCACTCCTCGCGTCACTGGGGCCTTTGGGTTCGCTAGCTCGCTCATCG<br>CCTGTTACCAGTTGCTTCCAGCCCCAGCCAAATCCAAGTGA
PEX11C-4A	XM_037567661.1	ATGGCCTCAGTGGACACCGTCAGAGGGGATCTTGGCCTGGTTGTTTTGTA<br>CCTAAGCAAGGCTGAGGCAAGAGATAAGATCTGTAGGGCTATACAATATGG<br>ATCCAAGTTCTTGAGCAACGGACAGCCAGGACCTGCACAGAATGTCGACA<br>AATCAACTAGTCTAGCTCGGAAAGTTTTCCGCCTGTTTAAGTTTGTTAATGA<br>TCTCCAAGCTCTGATTAGTCCCCCTGCCAAAGGAACTCCACTTCCACTGAT<br>CTTACTTGGAAAGTCGAAGAACGCGATGCTGTCAACTTTCCTCTTTCTGGA<br>CCAAATTGTTTGGGCTGGGAGAACAGGAGTATACAAGAACAAGGAGCGAG<br>CGGAGTTCCTTGGCAGGATAGCATTTTATTGCTTTCTCGGATCTAATACCTG<br>TACTACCATCATCGAGCTAGCGGAGCTTCAGCGGCTTTCCAAATCGATGAA<br>GAAGTTAGAGAAGGACCTCAAGCACCAAGAGCTGCACAAGAACGAGCAGT<br>ATCGGATGAAGCTGCAGAAGTCCAACGAGAGGCTGCTCGCCCTCATCAAA<br>TCGAGCCTCGACATAGTTGTTGCGGTGGGGCTCCTGCAACTTGCCCCGAA<br>AAAGGTCACTCCTCGCGTCACAGGGGCCTTTGGGTTCGCTAGCTCGCTCA<br>TCGCCTGTTACCAGTTGCTTCCAGCCCCAGCCAGATCCAAGTGA
CAT1-5A	MK091459.1	ATGGACCCCTACAAGTACCGGCCGTCGAGCTCCTTCAACGCCCCGATGTG<br>GAGCACCAACTCCGGCGCGCCCGTCTGGAACAACGACAACTCCCTCACC<br>GTCGGATCCCGAGGCCCGATCCTGCTGGAGGACTACCACCTGGTGGAGA<br>AGATCGCCGACTTCGACCGCGAGCGCATCCCGGAGCGGGTGGTGCACGC<br>CCGGGGCGCCAGCGCCAAGGGCTTCTTCGAGGTGACCCACGACGTCTCC<br>CACCTGACCTGCGCCGACTTCCTCCGCGCGCCGGGGGTGCAGACCCCC<br>GTCATCGTGCGCTTCTCCACCGTGATCCACGAGCGCGGCTCCCCGGAGA<br>CGCTCCGCGACCCGCGCGGCTTCGCCATCAAGTTCTACACCCGGGAGGG<br>CAACTGGGACCTGGTCGGCAACAACTTCCCCGTCTTCTTCATCCGCGACG<br>GCATGAAGTTCCCGGACATGGTGCACGCGCTCAAGCCCAACCCCAAGACC<br>CACATCCAGGAGAACTGGCGCATCCTCGACTTCTTCTCCCACCACCCGGA<br>GTCGCTCCACATGTTCACCTTCCTCTTCGACGACGTGGGCGTGCCCGCCG<br>ACTACCGCCACATGGACGGCTCCGGCGTCAACACCTACACGCTCGTCAAC<br>CGCGCCGGCAAGGCGCACTACGTCAAGTTCCACTGGAAGCCCACCTGCG<br>GCGTCAAGTCCCTCCTCGAGGAGGAGGCGGTCACCGTGGGCGGCGCCA<br>ACCACAGCCACGCCACCAAGGACCTGACCGGCTCCATCGCCGCCGGCAA<br>CTACCCGGAGTGGACGTTCTACATCCAGACCATCGACCCGGACCACGAGG<br>ATCGGTTCGACTTCGACCCGCTGGACGTGACCAAGACGTGGCCCGAGGA<br>CGTGGTGCCGCTGCAGCCGGTGGGGCGGCTGGTGCTGAACCGCAACATC

		GACAACTTCTTCGCGGAGAACGAGCAGCTCCCCTTCTGCCCCGGGATCAT<br>CGTCCCCGGGGTGTACTACTCGGACGACAAGCTGCTGCAGACGAGGATCT<br>TCTCCTACTCGGACACGCAGCGCCACCGGCTGGGGCCGAATTACCTGCTG<br>CTGCCGGCCAACGCGCCCAAGTGCTCCCACCACAACAACCACTACGACG<br>GGCTCATGAACTTCATGCACCGCGACGAGGAGGTCGACTACTTCCCCTCC<br>AGGTTCGACCCCGCCAAGCACGCGCCCCGCTACCCCATCCCCTCCCGCA<br>CCCTCAACGGCCGCCGCGAGAAGATGGTGATCGAGAAGGAGAACAACTT<br>CAAGCAGCCCGGGGAGAGGTACCGCTCCATGGACCCGGCAAGGCAAGAG<br>CGATTCATCAACAGATGGATCGATGCGCTGTCCGACCCCCGCCTCACCCA<br>CGAGATCAAGGCCATCTGGCTCTCCTACTGGTCTCAGGCTGACAAGTCTC<br>TCGGCCAGAAGCTCGCGAGCCGTCTCAGCTCCAAGCCGAGCATGTAA
CAT1-4B	D86327.1	ATGTGGAGCACCAACTCCGGCGCGCCCGTCTGGAACAACGACAACTCCC<br>TCACCGTCGGATCCCGAGGTCCGATCCTGCTGGAGGACTACCACCTGGT<br>GGAGAAGATCGCCGACTTCGACCGTGAGCGCATCCCGGAGCGGGTGGTA<br>CACGCCCGGGGCGCCACGGCCAAGGGCTTCTTCGAGGTCACCCACGAC<br>GTCTCCCACCTGACCTGCGCCGACTTCCTCCGCGCGCCGGGGGTGCAGA<br>CCCCCGTCATAGTGCGCTTCTCCACCGTGATCCACGAGCGCGGCTCCCC<br>CGAGACGCTCCGCGACCCGCGCGGCTTCGCCATCAAGTTCTACACCCGG<br>GAGGGCAACTGGGACCTGGTGGGCAACAACTTCCCCGTCTTCTTCATCCG<br>CGACGGCATGAAGTTCCCGGACATGGTGCACGCGCTCAAGCCCAACCCC<br>AAGACCCACATCCAGGAGAACTGGCGGATCCTCGACTTCTTCTCCCACCA<br>CCCGGAGTCGCTCCACATGTTCACCTTCCTCTTCGACGACATCGGCGTGC<br>CCGCCGACTACCGCCACATGGACGGCTCCGGCGTCAACACCTACACGCT<br>GGTGAACCGCGCCGGCAAGGCGCACTACGTCAAGTTCCACTGGAAGCCC<br>ACCTGCGGCGTCAAGTCGCTGCTGGAGGAGGAGGCGGTCACGGTGGGC<br>GGCACCAACCACAGCCACGCCACCAAGGACCTCACCGACTCCATCGCCG<br>CCGGCAACTACCCGGAGTGGACCTTCTACATCCAGACCATCGACCCGGA<br>CTATGAGGAGCGGTTCGACTTCGACCCGCTGGACGTGACCAAGACGTGG<br>CCCGAGGACGTGGTGCCGCTGCAGCCCGTGGGGCGGCTGGTGCTGAAC<br>CGCAACATCGACAACTTCTTCTCGGAGAACGAGCAGCTGGCCTTCTGCCC<br>CGGGATCATCGTCCCCGGGGTGTACTACTCGGACGACAAGCTGCTGCAG<br>ACAAGGATCTTCTCCTACTCCGACACGCAGCGCCACCGTCTGGGGCCCAA<br>CTACCTGCTGCTGCCGGCCAACGCGCCCAAGTGCTCCCACCACAACAAC<br>CACTACGACGGGCTCATGAACTTCATGCACCGCGACGAGGAGGTCGACT<br>ACTTCCCCTCAAGGTTCGACCCCGCCAAGCACGCGCCCCGCTACCCCAT<br>CCCCTCCCGCACCCTCAACGGCCGCCGCGAGAAGATGGTGATCGAGAAG<br>GAGAACAACTTCAAGCAGCCCGGGGAGAGGTACCGCTCCATGGACCCGG<br>CAAGGCAAGAGCGATTCATCAACAGATGGATCGACGCGCTGTCGGACCC<br>CCGCCTCACCCATGAGATCAAGGCCATCTGGCTCTCCTACTGGTCTCAGG<br>CTGACAAGTCTCTCGGCCAGAAGCTCGCGAGCCGTCTCAGCTCCAAGCC<br>GAGCATGTAA
CAT1-4D	AK454435.1	ATGTGGAGCACCAACTCCGGCGCGCCCGTCTGGAACAACGACAACTCCCT<br>CACCGTCGGATCCCGAGGTCCCATCCTGCTGGAGGACTACCACCTGGTG<br>GAGAAGATCGCCGACTTCGACCGCGAGCGCATCCCGGAGCGGGTGGTGC<br>ACGCGCGTGGCGCCAGCGCCAAGGGCTTCTTCGAGGTGACCCACGACGT<br>GTCCCACCTGACCTGCGCCGACTTCCTCCGCGCCCCCGGCGTGCAGACC<br>CCCGTCATCGTGCGCTTCTCCACCGTGATCCACGAGCGCGGCTCCCCCG<br>AGACGCTCCGCGACCCGCGCGGCTTCGCCATCAAGTTCTACACCCGGGA<br>GGGCAACTGGGACCTGGTCGGCAACAACTTCCCCGTCTTCTTCATCCGCG<br>ACGGCATGAAGTTCCCGGACATGGTGCACGCGCTCAAGCCCAACCCCAA<br>GACCCACATCCAGGAGAACTGGCGCATCCTCGACTTCTTCTCCCACCACC<br>CGGAGTCGCTCCACATGTTCACCTTCCTCTTCGACGACATCGGCGTGCCC<br>GCCGACTACCGCCACATGGACGGCTCCGGCGTCAACACCTACACGCTCGT<br>CAACCGCGCCGGCAAGGCGCACTACGTCAAGTTCCACTGGAAGCCCACC<br>TGCGGCGTCAAGTCGCTCCTCGAGGAGGAGGCGGTCACGGTGGGCGGC<br>ACCAACCACAGCCACGCCACCAAGGACCTGACCGACTCCATCGCCGCCG<br>GCAACTACCCGGAGTGGACCTTCTACATCCAGACCATCGACCCGGACCAC<br>GAGGACCGCTTCGACTTTGACCCGCTGGACGTGACCAAGACGTGGCCCG<br>AGGACGTGGTGCCGCTGCAGCCGGTGGGGCGGCTGGTGCTCAACCGCA<br>ACATCGACAACTTCTTCGCGGAGAACGAGCAGCTGGCCTTCTGCCCCGGG<br>ATCATCGTCCCCGGGGTGTACTACTCGGACGACAAGCTGCTGCAGACGAG<br>GATCTTCTCCTACTCGGACACGCAGCGCCACCGCCTGGGGCCCAACTACC

		TGCTGCTGCCGGCCAATGCGCCCAAGTGCTCCCACCACAACAACCACTAC GACGGGCTCATGAACTTCATGCACCGCGACGAGGAGGTCGACTACTTCCC CTCCAGGTTCGACCCCGCCAAGCACGCGCCCCGCTACCCCATCCCCTCG CGCACCCTCAACGGCCGCCGCGAGAAGATGGTGATCGAGAAGGAGAACA ACTTCAAGCAGCCCGGGGAGAGGTACCGCTCCATGGACCCGGCAAGGCA AGAGCGATTCATCAACAGATGGATCGATGCGCTGTCCGACCCCCGCCTCA CCCACGAGATCAAGGCCATCTGGCTCTCCTACTGGTCTCAGGCTGACAAG TCTCTCGGCCAGAAGCTCGCGAGCCGTCTCAGCTCGAAGCCGAGCATGTA A
CAT2-6A	XM_037594016.1	ATGGATCCCTGCAAGTTCCGGCCGTCGAGCAGCTTCGACACCAAGACGA CGACGACCAACGCCGGCCAGCCGGTGTGGAACGACAACGAGGCGCTCA CCGTCGGCCCGCGCGGCCCGATCTTGCTGGAGGACTACCACCTGCTGGA GAAGATCGCCCACTTCGCGCGCGAGCGCATCCCGGAGCGGGTGGTGCA CGCGCGGGGCGCCTCCGCCAAGGGCTTCTTCGAGTGCACCCACGACGTG ACGGGCTTGACCTGCGCCGACTTCCTCCGCGCGCCGGGGGCGCGCACG CCGGTCATCGTCCGCTTTTCCACGGTGATCCACGAGCGCGGCTCGCCGG AGACGATCCGTGACCCGCGCGGGTTCGCCGTCAAGTTCTACACGCGCGA GGGCAACTGGGACCTGCTCGGCAACAACTTCCCCGTCTTCTTCATCCGCG ACGGCATCAAGTTCCCCGACGTCATCCACGCCTTCAAACCCAACCCCAAG TCGCATGTGCAGGAGTACTGGCGCGTCTTCGACTTCCTCTCCCACCACCC CGAGAGCCTCCACACCTTCTTCTTCCTCTTCGACGACGTCGGCATCCCCA CCGACTACCGCCACATGGACGGCTTCGGGGTCAACACCTACACCTTCGTC TCCCGTGCCGGCAAGTCCCACTACGTCAAGTTCCACTGGCGCCCCACCT GCGGCGTCAGCTGCCTCATGGACGACGAGGCCACGCTCGTCGGCGGCA AGAACCACAGCCACGCCACGCAGGACCTCTACGACTCCATCGACGCCGG CAACTTCCCCGAGTGGAAGCTGTTCGTGCAGGTCATCGACCCCGACGAC GAGGACCGCTTCGACTTCGACCCGCTCGACGACACCAAGACATGGCCCG AGGACCTCGTCCCCCTCCAGCCCGTCGGCCGCCTCGTGCTCGACCGCAA CGTCGACAACTTCTTCAACGAGAACGAGCAGCTGGCCTTCGGCCCGGGG CTCGTCGTGCCCGGGATCTACTACTCCGACGACAAGATGCTGCAGTGCAG GGTGTTCGCCTACGCCGACACGCAGCGCTACCGCCTCGGCCCAAACTAC CTGATGCTCCCCGTCAACGCGCCCAAGTGCGGCTTCAAGAACAACCACTA CGACGGCGCCATGAACTTCATGCACCGCGACGAGGAGGTGGACTACTAC CCGTCCCGCCACGCGCCGCTCCGGCAGGCCGAGCCGGCCAGCTTCCCC GTGCCGACGAGGCCCGTCGTCGGGAAAAGGGAGAAGACGAGGATCAAGA AGGAGAACGACTTCGTGCAGCCCGGGGAGAGGTACCGCAGCTGGGCGC CCGACCGCCAGGACAGGTTCGTGCGCCGCTTCGCCGACGCCCTCGCCCA CCCCAAGGTCAGCCACGAGCTCCGCGTCATCTGGATCGACTTCCTCTCCA AGTGTGACAAGTCGTGCGGCATGAAGGTGGCCAACCGCCTTAACGTGAA GCCAAGCATGTGA
CAT2-6B	XM_037599040.1	ATGGATCCCTGCAAGTTCCGGCCGTCGAGCAGCTTCGACACCAAGACGA CGACGACGAACGCCGGCCAGCCCGTGTGGAACGACAACGAGGCGCTCA CCGTCGGCCCCCGCGGCCCCATCCTGCTGGAGGACTACCACCTGCTGGA GAAGATCGCCCACTTCGCGCGCGAGCGCATCCCGGAGCGGGTGGTGCA CGCCCGGGGCGCCTCCGCCAAGGGCTTCTTCGAGTGCACCCACGACGTC ACCGGCCTCACCTGCGCCGACTTCCTCCGCGCGCCGGGGGCGCGGACG CCCGTCATCGTCCGCTTCTCCACCGTCATCCACGAGCGCGGCTCGCCCG AGACGATCCGCGACCCGCGCGGGTTCGCCGTCAAGTTCTACACGCGCGA GGGCAACTGGGACCTGCTCGGCAACAACTTCCCCGTCTTCTTCATCCGCG ACGGCATCAAGTTCCCCGACGTCATCCACGCCTTCAAGCCCAACCCCAAG TCCCACGTGCAGGAGTACTGGCGCGTCTTCGACTTCCTCTCCCACCACCC CGAGAGCCTCCACACCTTCTTCTTCCTCTTCGACGACGTCGGCATCCCCA CGGACTACCGCCATATGGACGGCTTCGGCGTCAACACCTACACCTTCGTC TCCCGCGCCGGCAAGGCCCACTACGTCAAGTTCCACTGGCGCCCCACCT GCGGCGTCAGCTGCCTCATGGACGACGAGGCCACCCTCGTCGGCGGCAA GAACCACAGCCACGCCACCCAGGACCTCTACGACTCCATCGACGCCGGC AACTTCCCCGAGTGGAAGCTCTTCGTGCAGGTCATCGACCCCGACGACGA GGACCGCTTCGACTTCGACCCGCTCGACGACACCAAGACTTGGCCCGAG GACCTCGTCCCCCTCCAGCCCGTGGGACGCCTCGTGCTCGACCGCAACG TCGACAACTTCTTCAACGAGAACGAGCAGCTCGCCTTCGGCCCCGGCCTC GTCGTGCCCGGGATCTACTACTCCGACGACAAGATGCTGCAGTGCAGGG TGTTCGCCTACGCCGACACGCAGCGCTACCGCCTGGGGCCAAACTACCT

		GATGCTCCCCGTCAACGCGCCCAAGTGCGGCTTCAAGAACAACCACTACG<br>ACGGCGCCATGAACTTCATGCACCGGGACGAGGAGGTGGACTACTACCC<br>GTCCCGCCACGCGCCGCTCCGGCACGCTGAGCCGGCCAGCTTCCCCGT<br>GCCGACGCGGCCCGTCGTCGGGAAAAGGGAGAAGACGAGGATCAAGAA<br>GGAGAACGACTTCGTGCAGCCCGGGGAGAGGTACCGCAGCTGGGCGCC<br>CGACCGGCAGGACAGGTTCGTGCGCCGCTTCGCCGACGCGCTCGCCCA<br>CCCCAAGGTCAGCCACGAGCTCCGCGTCATCTGGATCGACTTCCTCTCCA<br>AGTGTGACAAGTCGTGCGGCATGAAGGTGGCCAACCGTCTCAACGTGAA<br>GCCAAGCATGTGA
CAT2-6D	AK456618.1	ATGGATCCCTGCAAGTTCCGGCCGTCGAGCAGCTTCGACACCAAGACGAC<br>GACCACCAACGCCGGCCAGCCGGTGTGGAACGACAACGAGGCGCTCACC<br>GTCGGCCCGCGCGGCCCCATCCTGCTGGAGGACTACCACCTGCTGGAGA<br>AGATCGCCCACTTCGCGCGCGAGCGCATCCCGGAGCGCGTCGTCCACGC<br>CAGGGGCGCCTCCGCCAAGGGCTTCTTCGAGTGCACCCACGACGTCACC<br>GGCCTCACCTGCGCCGACTTCCTCCGCGCGCCCGGGGCGCGCACCCCC<br>GTCATCGTCCGCTTCTCCACCGTCATCCACGAGCGCGGCTCGCCCGAGAC<br>CATCCGCGACCCGCGCGGGTTCGCCGTCAAGTTCTACACGCGCGAGGGC<br>AACTGGGACCTGCTCGGCAACAACTTCCCCGTCTTCTTCATCCGCGACGG<br>CATCAAGTTCCCCGACGTCATCCACGCCTTCAAGCCCAACCCCAAGTCGC<br>ACGTGCAGGAGTACTGGCGCGTCTTCGACTTCCTCTCCCACCACCCGGAG<br>AGCCTCCACACCTTCTTCTTCCTCTTCGACGACGTCGGCATCCCCACCGA<br>CTACCGCCACATGGACGGCTTCGGCGTCAACACCTACACCTTCGTCTCCC<br>GCGCTGGCAAGTCCCACTACGTCAAGTTCCACTGGAGGCCCACCTGCGG<br>CGTCAGCTGCCTCATGGACGACGAGGCCACCCTCGTCGGCGGCAAGAAT<br>CACAGCCACGCCACCCAGGACCTCTACGACTCCATCGACGCCGGCAACTT<br>CCCCGAGTGGAAGCTCTTCGTGCAGGTCATCGACCCCGACGACGAGGAC<br>CGCTTCGACTTCGACCCGCTCGACGACACCAAGACCTGGCCCGAGGACC<br>TCGTCCCCCTCCAGCCCGTGGGGCGCCTCGTGCTCGACCGCAACGTCGA<br>CAACTTCTTCAACGAGAACGAGCAGCTGGCCTTCGGCCCGGGGCTCGTC<br>GTGCCCGGGATCTACTACTCGGACGACAAGATGCTGCAGTGCAGGGTGTT<br>CGCCTACGCCGACACGCAGCGCTACCGCCTGGGGCCCAACTACCTGATG<br>CTCCCCGTCAACGCGCCCAAGTGCGGCTTCAAGAACAACCACTACGACGG<br>CGCCATGAACTTCATGCACCGCGACGAGGAGGTGGACTACTACCCGTCAC<br>GCCACGCGCCGCTCCGGCACGCCGAGCCGGCCAGCTTCCCCGTGCCCA<br>CGCGGCCCGTCGTCGGAAAAAGGGAGAAGACGAGGATCAAGAAGGAGAA<br>CGACTTCGTGCAGCCCGGGGAGAGGTACCGCAGCTGGGCGCCCGACCG<br>CCAGGACAGGTTCGTGCGCCGCTTCGCCGACGCGCTCGCCCACCCCAAG<br>GTCAGCCACGAGCTCCGCGTCATCTGGATCGACTTCCTCTCCAAGTGTGA<br>CAAGTCGTGCGGCATGAAGGTGGCCAACCGCCTTAACGTGAAGCCAAGCA<br>TGTGA

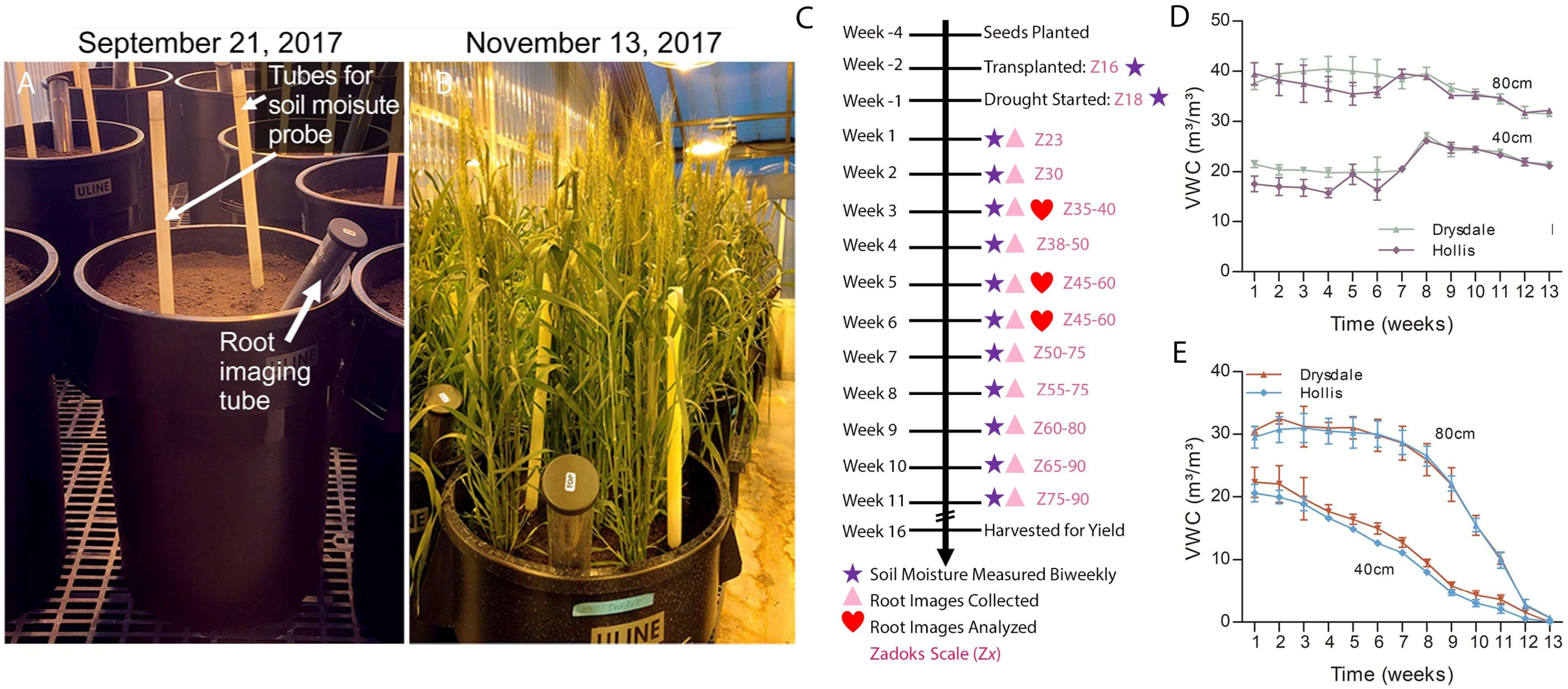

Supplemental Figure S1. Experimental setup for measuring impact of drought on root growth and yield.

A, A representative bin prior to planting the seedlings. Each bin contains one root imaging tube in the center and two soil moisture probe tubes on either side of the imaging tube.

B, A representative image a watered control bin with Drysdale plants. Each bin contained five plants.

C, The experimental timeline. The seedlings were acclimated to the bins for two weeks. Drought was induced by stopping watering and maintained until the harvest. Control bins were watered daily until the plants started to dry. Soil moisture was measured twice per weak. Root images were collected once a week, but only data from wheat 3, 5, and 6 were analyzed. Yield parameters were measured at the end of the experiment.

D, E, The average soil VWC values in watered control (**D**) and drought (**E**) bins taken at 40cm or 80cm soil depth. The error bars represent standard deviation of values from two watered control bins or three drought stress bins.

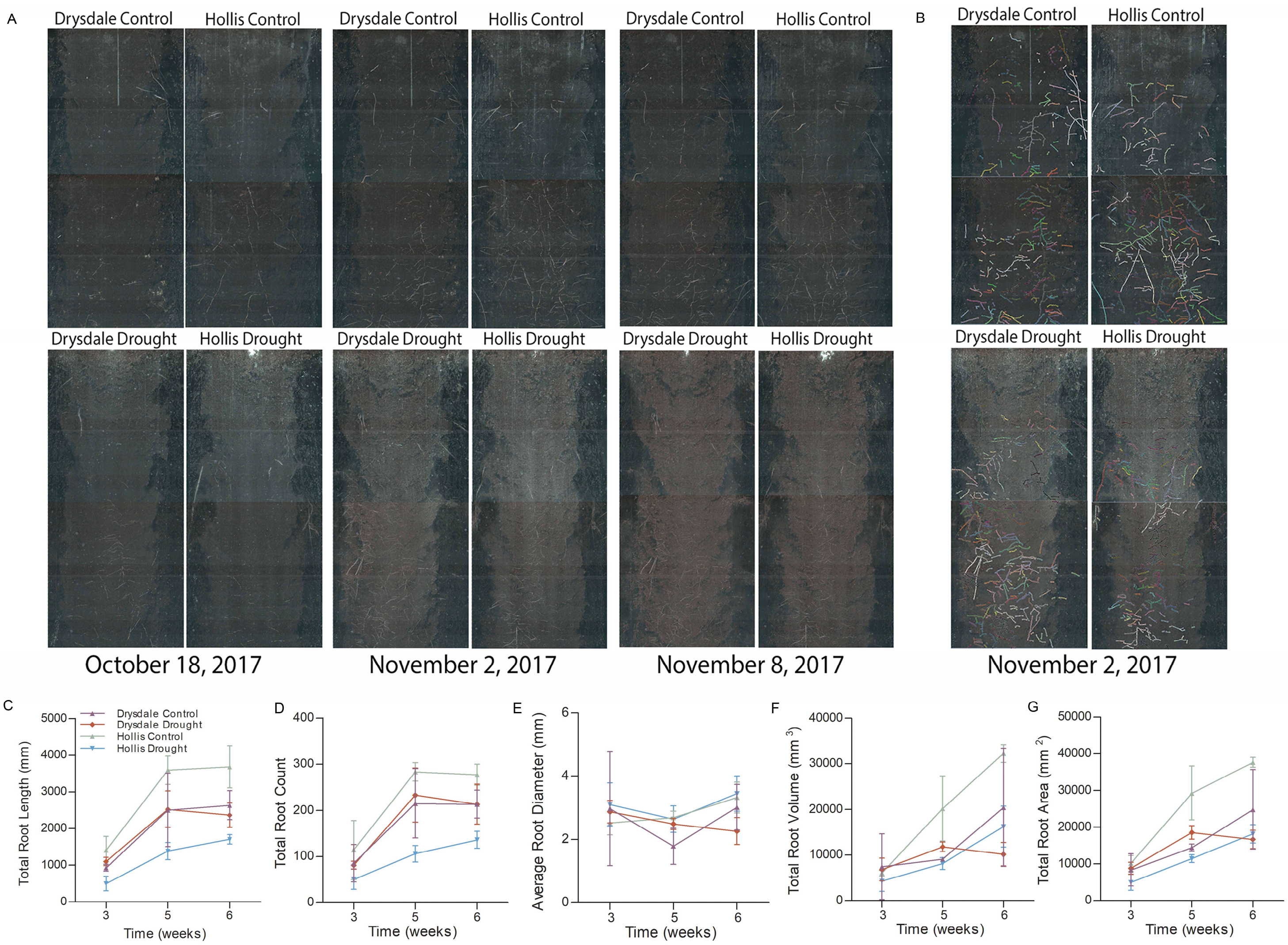

Supplemental Figure S2. Analysis of root growth response to drought stress.

A, Representative images of roots in control and drought stress bins for Drysdale and Hollis for each of the 3 timepoints analyzed.

B, Representative images of root analysis using RootSnap! Program for control and drought stress bins.

C, **D**, **E**, **F**, **G**, The average total root length (**C**), the average total root count (**D**), the average root diameter (**E**), the average root volume (**F**), and the average total root area (**G**), for control and drought stressed Drysdale and Hollis plants. The error bars represent standard deviation of values from two watered control bins or three drought stress bins.

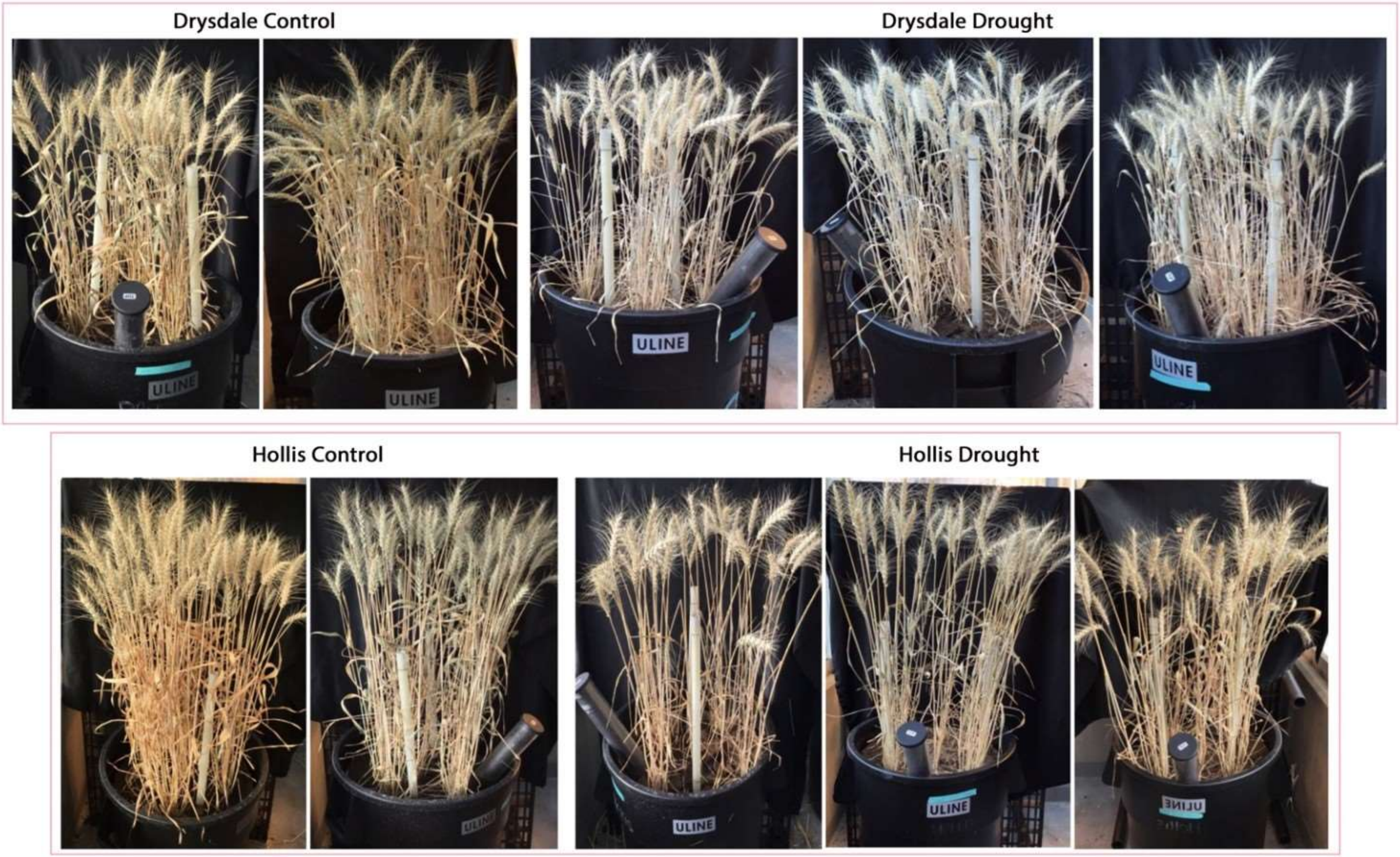

Supplemental Figure S3. Images of bins taken prior to harvest.

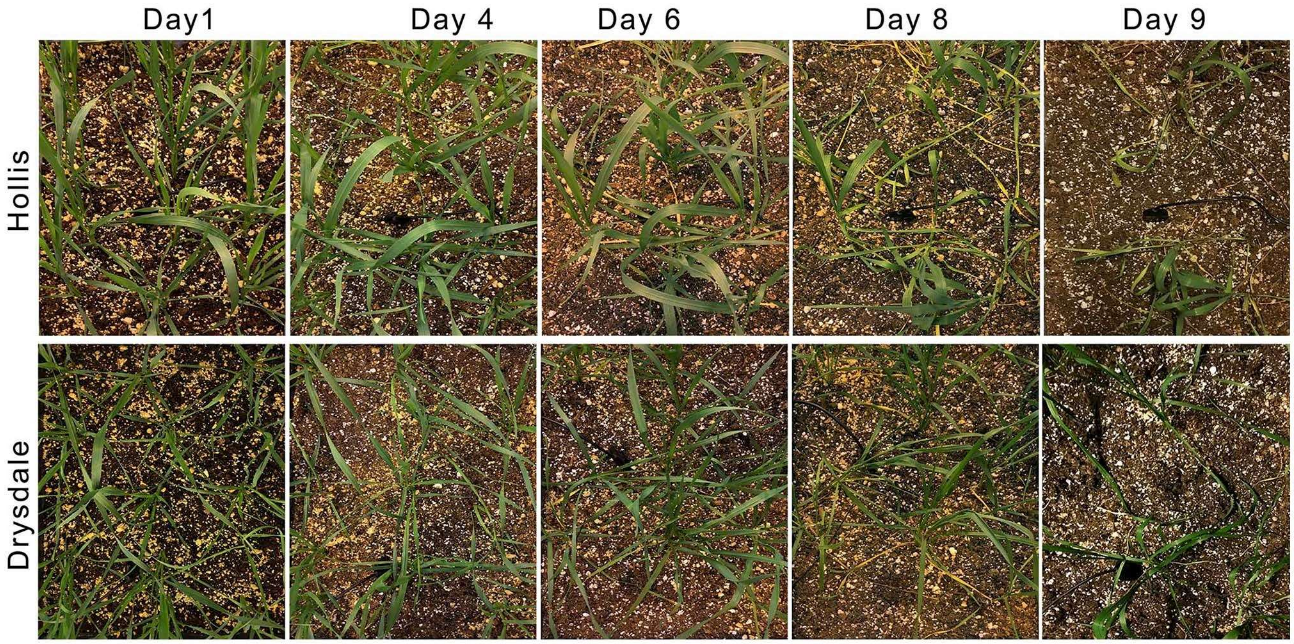

Supplemental Figure S4. Representative images of Hollis and Drysdale plants taken at different stages of drought stress.

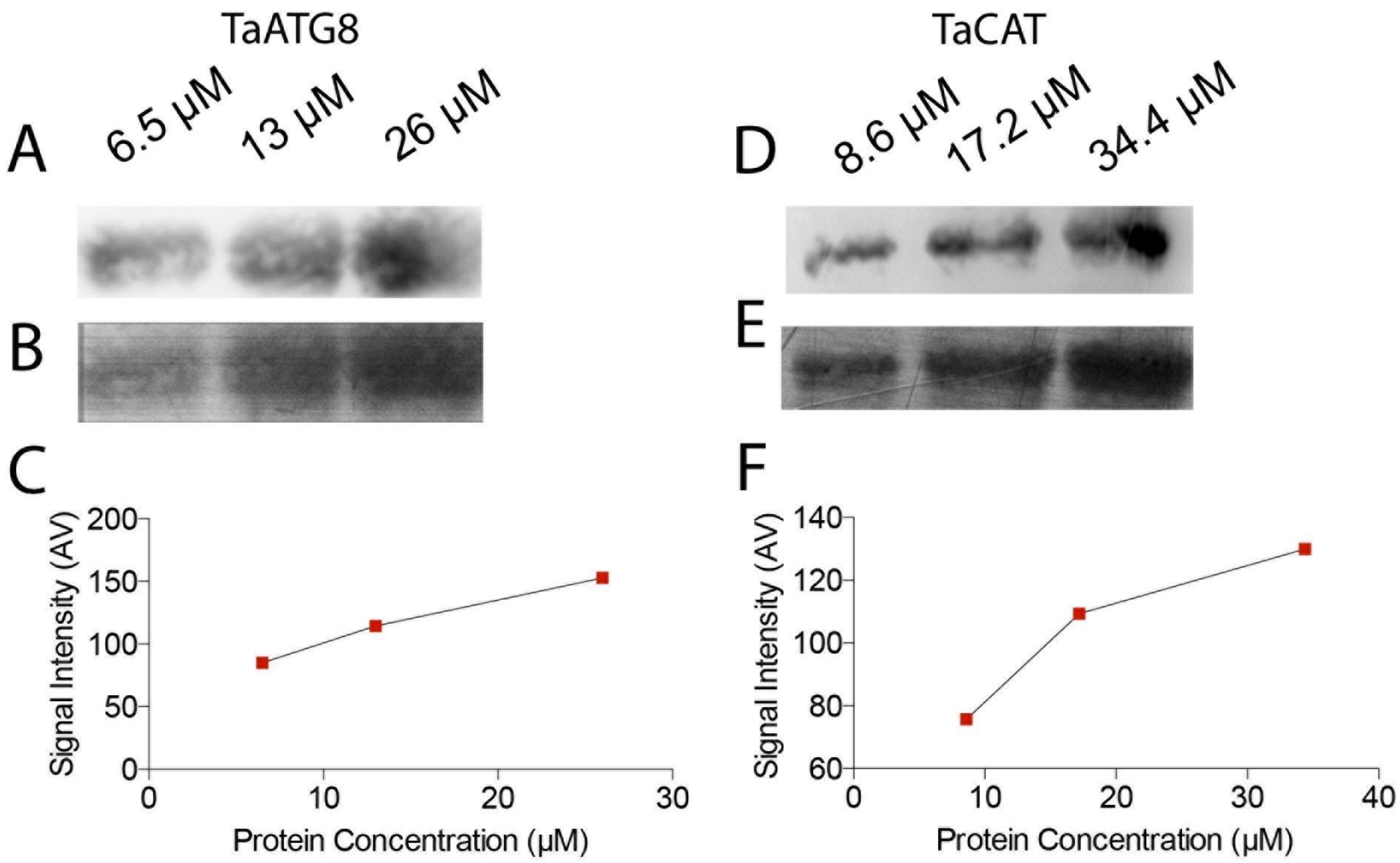

Supplemental Figure S5. Correlation between protein loading and Western blotting signal for wheat ATG-8 and CAT antibodies.

A,**D** Western blotting with anti-ATG8 or anti-CAT at different protein loading (μM).

B,**E**, Colloidal silver staining of the nitrocellulose membrane shown in **A** or **D**.

C,**F**, Quantification of the luminescence signal in **A** or **D**.
